# Type I Interferons Suppress Anti-parasitic Immunity and Can Be Targeted to Improve Treatment of Visceral Leishmaniasis

**DOI:** 10.1016/j.celrep.2020.01.099

**Published:** 2020-02-25

**Authors:** Rajiv Kumar, Patrick T. Bunn, Siddharth Sankar Singh, Susanna S. Ng, Marcela Montes de Oca, Fabian De Labastida Rivera, Shashi Bhushan Chauhan, Neetu Singh, Rebecca J. Faleiro, Chelsea L. Edwards, Teija C.M. Frame, Meru Sheel, Rebecca J. Austin, Steven W. Lane, Tobias Bald, Mark J. Smyth, Geoffrey.R. Hill, Shannon E. Best, Ashraful Haque, Dillon Corvino, Nic Waddell, Lambross Koufariotis, Pamela Mukhopadhay, Madhukar Rai, Jaya Chakravarty, Om Prakash Singh, David Sacks, Susanne Nylen, Jude Uzonna, Shyam Sundar, Christian R. Engwerda

**Affiliations:** 1 Centre of Experimental Medicine and Surgery, Institute of Medical Sciences, Banaras Hindu University, Varanasi, UP, India; 2 Department of Medicine, Institute of Medical Sciences, Banaras Hindu University, Varanasi, UP, India; 3 QIMR Berghofer Medical Research Institute, Brisbane, QLD, Australia; 4 Griffith University, Institute of Glycomics, Gold Coast, QLD, Australia; 5 Griffith University, School of Natural Sciences, Nathan, QLD, Australia; 6 University of Queensland, School of Medicine, Brisbane, QLD Australia; 7 National Centre for Epidemiology and Population Health, Research School of Population Health, College of Health and Medicine, The Australian National University, Canberra, ACT, Australia; 8 Clinical Research Division, Fred Hutchinson Cancer Research Center, Seattle, WA, USA; 9 National Institute of Allergy and Infectious Diseases, NIH, Bethesda, MD, USA; 10 Department of Microbiology, Tumor and Cell Biology, Karolinska Institute, Stockholm, Sweden; 11 Department of Immunology, Max Rady College of Medicine, University of Manitoba, Winnipeg, MB, Canada; 12 These authors contributed equally; 13 Senior author; 14 Lead Contact

## Abstract

Type I interferons (IFNs) play critical roles in anti-viral and anti-tumor immunity. However, they also suppress protective immune responses in some infectious diseases. Here, we identify type I IFNs as major upstream regulators of CD4^+^ T cells from visceral leishmaniasis (VL) patients. Furthermore, we report that mice deficient in type I IFN signaling have significantly improved control of *Leishmania donovani*, a causative agent of human VL, associated with enhanced IFNγ but reduced IL-10 production by parasite-specific CD4^+^ T cells. Importantly, we identify a small-molecule inhibitor that can be used to block type I IFN signaling during established infection and acts synergistically with conventional anti-parasitic drugs to improve parasite clearance and enhance anti-parasitic CD4^+^ T cell responses in mice and humans. Thus, manipulation of type I IFN signaling is a promising strategy for improving disease outcome in VL patients.

## In Brief

CD4^+^ T cells are critical for control of intracellular parasites such as *Leishmania donovani*. Kumar et al. show that type I interferons (IFNs) suppress Th1 cells and promote IL-10-producing CD4^+^ T cells during visceral leishmaniasis (VL). Thus, manipulation of type I IFN signaling may improve disease outcome in VL patients.

## Graphical Abstract

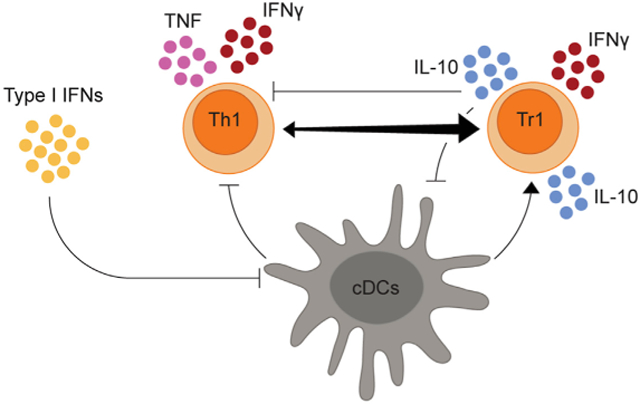

## INTRODUCTION

Visceral leishmaniasis (VL) is a chronic infectious disease caused by the protozoan parasites *Leishmania donovani* and *L. infantum* (*chagasi*) (Singh et al., 2016). There were between 50,000 and 90,000 clinical cases in 2017 (Burza et al., 2018), down from 200,000–400,000 cases in 2012 (Alvar et al., 2012), reflecting major efforts in the past decade to control this disease in the Indian subcontinent (Singh et al., 2016). However, VL still remains a major threat to human health in many other parts of the world, most notably in East Africa (Burza et al., 2018). Furthermore, given the relatively high frequency of asymptomatic individuals relative to those that develop VL (Singh et al., 2014) and that drug-cured VL patients still harbor live parasites (Sundar et al., 2014), re-emergence of the disease in previously endemic areas is an ongoing threat.

*L. donovani* invades and resides within tissue macrophages in the spleen, liver, and bone marrow (Kumar and Nylén, 2012). Experimental infection of C57BL/6 mice with *L. donovani* results in development of organ-specific pathology that shares features of fulminant disease manifested in humans (Stanley and Engwerda, 2007). In this context, the liver of an *L. donovani*-infected mouse is a site of resolving infection, reminiscent of parasite control in asymptomatic individuals. In contrast, parasites persist in the spleen and bone marrow, contributing to development of chronic disease similar to what is observed in VL patients (Engwerda and Kaye, 2000). VL is often characterized by locally dysregulated immune responses that impair parasite control at the expense of limiting tissue damage caused by excessive inflammation (Engwerda et al., 2014). CD4^+^ T cell responses are integral to disease outcome, with interferon γ (IFNγ)-producing, Tbet^+^ CD4^+^ T (Th1) cells critical for parasite clearance (Engwerda et al., 1998; Gorak et al., 1998; Stern et al., 1988; Taylor and Murray, 1997). However, many immunoregulatory molecules and pathways, most notably those associated with interleukin-10 (IL-10) production, are activated following infection, which can suppress anti-parasitic CD4^+^ T cell functions (Faleiro et al., 2014). These include Blimp-1-mediated induction of IL-10 production by Th1 (type 1 regulatory [Tr1]) cells during clinical and experimental VL (Montes de Oca et al., 2016a; Nylén et al., 2007).

Type I IFNs include IFNα and IFNβ family members that bind to a cell surface IFNα receptor (IFNαR) comprising IFNαR1 and IFNαR2 chains. They were first described in viral infection as critical effector cytokines required for viral clearance (Fensterl and Sen, 2009). However, contrary to their protective role in viral immunity, type I IFNs can exhibit immunoregulatory effects that impede control of some non-viral pathogens (Donovan et al., 2017; González-Navajas et al., 2012; Moreira-Teixeira et al., 2018; Silva-Barrios and Stäger, 2017). We previously reported that type I IFNs increased susceptibility to experimental cerebral malaria caused by *Plasmodium berghei* ANKA (PbA) (Haque et al., 2011). In this model, type I IFNs inhibited anti-*Plasmodium* Th1 cell responses via suppression of dendritic cell (DC)-mediated CD4^+^ T cell activation (Haque et al., 2014). Similar results were reported following experimental infection with the protozoan parasite *Trypanosoma cruzi*, where type I IFNs suppressed Th1 cell responses that controlled local parasite burdens (Chessler et al., 2011). We have also shown that type I IFNs suppress Th1 cell but enhance Tr1 cell responses in volunteers participating in controlled human malaria infection studies (Montes de Oca et al., 2016b). Type I IFNs have also been shown to promote anti-parasitic immunity in infection caused by *Toxoplasma gondii* (Orellana et al., 1991; Schmitz et al., 1989) and *P. yoelii* (Yu et al., 2016) as well as the late stages of *Trypanosoma brucei rhodesiense* (Lopez et al., 2008). Together, these data indicate distinct roles for this cytokine family in different parasitic infections, depending at least in part on pathogen inoculum, timing of experimental interventions, and/or kinetics of infection and progression to disease (Silva-Barrios and Stäger, 2017).

Leishmaniasis encompasses a spectrum of disease ranging from localized cutaneous lesions to visceralizing, systemic forms (Burza et al., 2018). The role of type I IFNs in this disease is still unclear and likely differs depending on the causative species and type of disease (Silva-Barrios and Stäger, 2017). In mouse models of cutaneous leishmaniasis, type I IFNs have positive and negative influences on disease outcome, depending on mouse strain and infecting species (Buxbaum, 2010; Khouri et al., 2009; Mattner et al., 2004; Xin et al., 2010). Interestingly, an endogenous virus found in *L. guyanensis* promoted type I IFN production by infected macrophages, causing reduced expression of IFNγ receptors associated with increased parasite growth and dissemination (Rossi et al., 2017). Early work on VL caused by *L. donovani* found that mice treated with the type I IFN inducer poly(I:C) 1 day prior to infection had improved control of parasite growth, whereas treatment during the course of infection exacerbated the disease (Herman and Baron, 1970). Type I IFN signaling to B cells has been shown more recently to stimulate endosomal Toll-like receptor (TLR) expression and cytokine production associated with inefficient control of splenic parasite growth (Silva-Barrios and Stäger, 2017). Knowledge about the role of type I IFNs in human VL is limited.

Type I IFN production and effects are highly context-specific regarding local tissue microenvironment and disease setting (Tough, 2012). Nearly all cells have the capacity to produce type I IFNs, including fibroblasts, endothelial cells, and leukocytes (González-Navajas et al., 2012). Previous work using models of viral infection reported an association between type I IFNs and the transcription factor signal transducer and activator of transcription 1 (STAT1). STAT1 is activated following recruitment of Janus-activated kinase (JAK) 1 and 2 to the type I IFN receptor (Platanias, 2005). STAT1 can mediate type I IFN-suppressive functions in these models via induction of IL-10 production and subsequent downregulation of IFNγ receptor on natural killer (NK) and T cells (Trinchieri, 2010). A similar immunosuppressive mechanism has been postulated in tuberculosis (Donovan et al., 2017; Moreira-Teixeira et al., 2018). However, the effect of type I IFNs on IL-10 production in parasitic disease is less clear (Chessler et al., 2011; Haque et al., 2011). Here we show that type I IFNs contribute to *L. donovani* persistence by suppressing Th1 cell development and promoting Tr1 cell expansion. Importantly, we also demonstrate the therapeutic potential of targeting type I IFN signaling to improve anti-parasitic immunity in VL patients.

## RESULTS

### Type I IFNs Are Important Upstream Regulators of CD4^+^ T Cells from VL Patients

**Figure 1.**
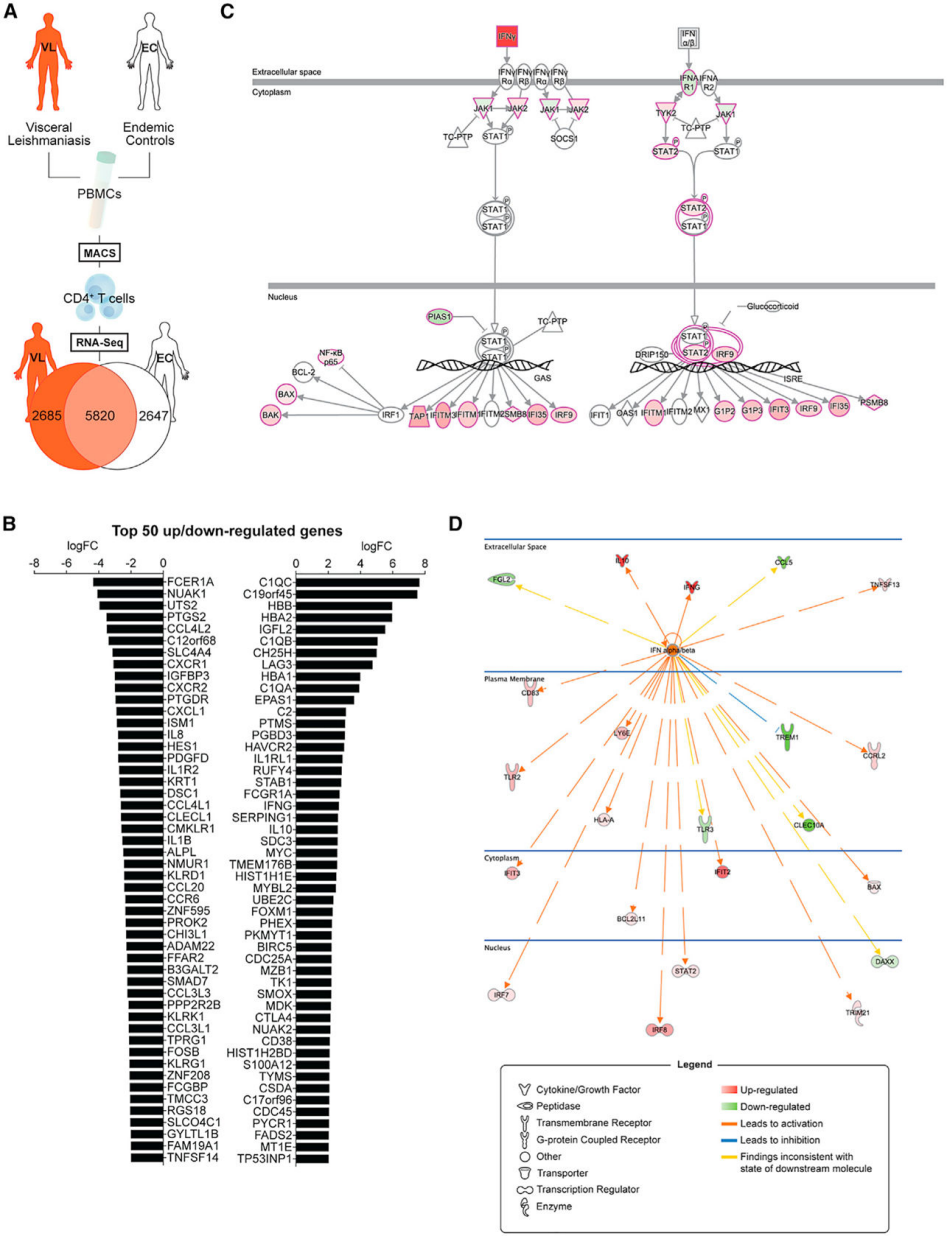
Type I IFNs Are Major Upstream Regulators of VL Patient CD4^+^ T Cell Responses (A) Schematic showing a brief outline of the workflow for isolating peripheral blood CD4^+^ T cells from visceral leishmaniasis (VL) patients (n = 12) and endemic controls (ECs; n = 12) for RNA-seq analysis and subsequent identification of differentially expressed genes indicated in the Venn diagram. (B) The top 50 up- and downregulated genes identified in VL patient CD4^+^ T cells relative to EC CD4^+^ T cells are listed. (C) The type I interferon (IFN) signaling pathway was identified as a canonical pathway (*Z* score of 2.828, p = 1.38 × 10^−4^) in CD4^+^ T cells from VL patients by Ingenuity Pathways Analysis. The pathway visualization shows upregulated (red) and downregulated (green) genes within the dataset, relative to CD4^+^ T cells from ECs. IFNα and IFNβ were found to be major upstream regulators (predicted activation state, activated; activation *Z* score of 2.490) of VL patient CD4^+^ T cells in this analysis. (D) The network shows all upregulated (red) and downregulated (green) genes within the dataset that were predicted to be regulated by IFNα and IFNβ and illustrates the predicted relationship between IFNα and IFNβ and these genes.

To identify factors that contribute to the inability of CD4^+^ T cells from VL patients to control parasite growth, we isolated CD4^+^ T cells from the blood of VL patients and endemic controls (ECs) (Table S1), prepared mRNA, and subjected samples to RNA sequencing (RNA-seq) to identify differentially expressed genes (Figure 1A; Table S2). The top 50 differentially upregulated genes in CD4^+^ T cells from VL patients, relative to ECs, included many identified in our recent focused analysis of 130 immune-related genes (Chauhan et al., 2019; Edwards et al., 2018), such as *IL10, IFNG, HAVCR2* (encoding TIM3), and *CTLA4*, as well as many previously unreported genes (Figure 1B). Notable downregulated genes in VL patient CD4^+^ T cells included *CXCR1, CXCR2, IL8, IL1B, CCL20*, and *CCR6* (Figure 1B).

**Figure 2.**
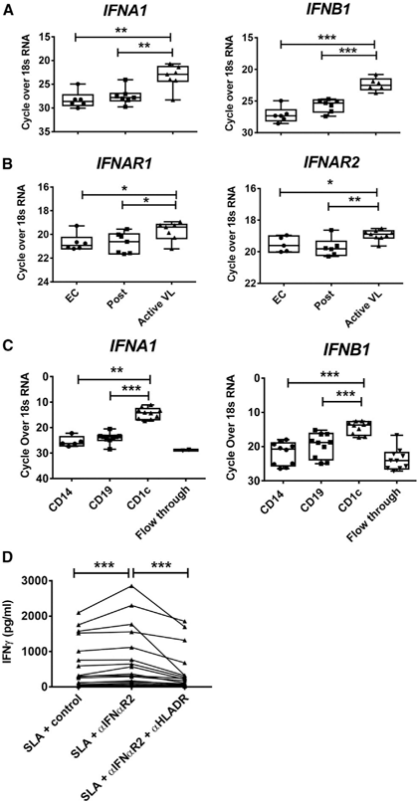
PBMCs from VL Patients Have a Type I IFN Gene Signature (A and B) RNA isolated from peripheral blood mononuclear cells (PBMCs) from VL patients upon admission to the clinic (active VL, n = 7–9), 3–6 months post-treatment (post, n = 6–7), and from ECs (n = 6) was subjected to qPCR to measure *IFNA1* and *IFNB1* (A) and *IFNAR1* and *IFNAR2* (B) mRNA transcripts. (C) *IFNA1* and *IFNB1* mRNA levels were also measured by qPCR in CD14^+^, CD19^+^, and CD1c^+^ cells isolated from VL patient PBMCs (n = 10) by magnetic activated cell sorting (MACS) as well as the flow through cells. (D) Antigen-specific IFNγ production was measured in whole-blood cells from VL patients upon admission to the clinic (n = 17), cultured for 24 h with soluble leishmania antigen (SLA) with control, anti-IFNαR1, or anti-HLA-DR antibodies as indicated. Median + minimum and maximum; *p < 0.05, **p < 0.01, and ***p < 0.001; significance assessed by Wilcoxon matched-pairs signed-rank test or Mann-Whitney test, as appropriate.

Pathways analysis identified canonical molecules related to both IFNγ and type I IFN signaling pathways (Figure 1C), and IFNα and IFNβ were recognized as major upstream regulators of CD4^+^ T cells from VL patients relative to ECs (Figure 1D). Given the known protective roles of IFNγ in VL (Kumar et al., 2014), we focused our attention on the lesser-known effects of type I IFNs. Examination of mRNA isolated from peripheral blood mononuclear cells (PBMCs) from VL patients before and after drug treatment, and ECs showed increased *IFNA1, IFNB1, IFNAR1*, and *IFNAR2* transcripts in VL patient cells before drug treatment relative to post-treatment and EC samples (Figures 2A and 2B). Furthermore, expression of *IFNA1* and *IFNB1* was highest in CD1c^+^ DCs relative to B cells (CD19^+^) and monocytes (CD14^+^) (Figure 2C), other likely cellular sources of these cytokines in experimental leishmaniasis (Rossi et al., 2017; Silva-Barrios et al., 2016). Limited expression of *IFNA1* and *IFNB1* was measured in the remaining PBMC populations (Figure 2C). Critically, when type I IFN signaling was blocked in an antigen-specific whole-blood assay (WBA) using samples from VL patients, we found increased IFNγ production in all 17 VL patient samples tested (Figure 2D). This increased IFNγ production was inhibited by blocking major histocompatibility complex (MHC) class II-mediated activation of CD4^+^ T cells in 15 of the 17 VL patient samples tested (Figure 2D). Thus, type I IFNs produced by VL patients are upstream regulators of CD4^+^ T cells and suppress antigen-specific IFNγ production.

### *Ifnar1^−/−^* Mice Show Improved Parasite Clearance and an Enhanced Inflammatory Cytokine Profile

**Figure 3.**
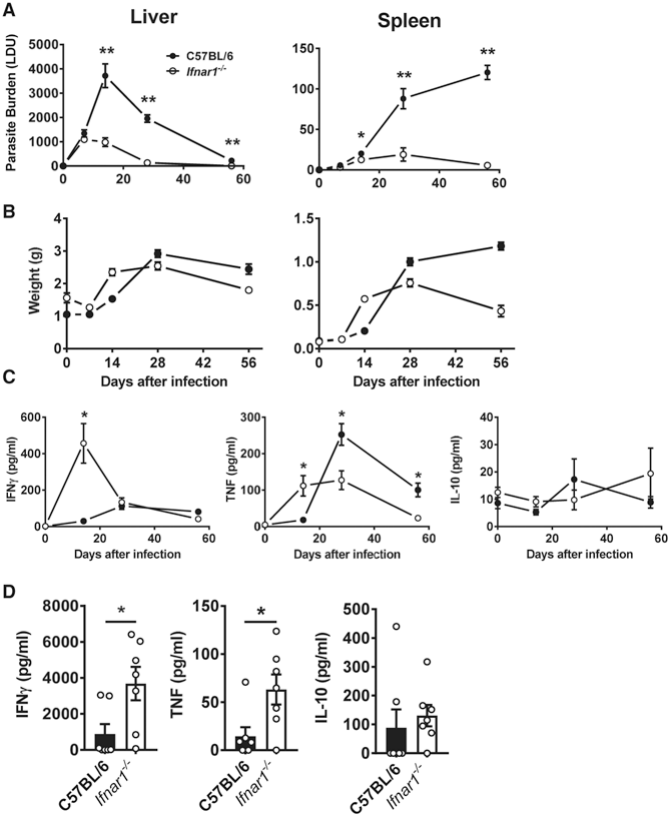
*Ifnar1*-Deficient Mice Have Improved Parasite Clearance and an Improved Inflammatory Response (A and B) *Ifnar1^−/−^* (open circles and columns) and control B6 WT (closed circles and columns) mice were infected with *L. donovani*, and liver and spleen parasite burdens (A) and organ weights (B) were measured on days 7, 14, 28, and 56 p.i., as indicated. (C and D) Serum cytokine (IFNγ, TNF, and IL-10) levels were also measured (C), as well as cytokine production by splenic mononuclear cells isolated from infected mice on day 14 p.i., after 72 h in culture with fixed *L. donovani* amastigotes (D). n = 4–7 mice per group. Each experiment was conducted 3–5 times. Mean ± SEM; *p < 0.05 and **p < 0.01; significance assessed by one-way ANOVA or Mann-Whitney test, as appropriate.

To better understand the role of type I IFN signaling during *L. donovani* infection, we employed an experimental model of VL where *Ifnar1^−/−^* mice were infected with *L. donovani* alongside B6 wild-type (WT) control mice. Splenic and hepatic parasite burdens were measured on days 7, 14, 28, and 56 post-infection (p.i.) (Figure 3). Strikingly, *Ifnar1^−/−^* mice exhibited significantly reduced parasite burdens in the liver and spleen over the course of infection compared with WT mice (Figure 3A). This difference was evident from day 14 p.i. onward, a time of infection primarily associated with infiltration and activation of effector T cell populations (Bunn et al., 2014; Murray et al., 1992). It should be noted that differences in splenic parasite burdens were not always evident this early and were not consistently observed until day 28 p.i. However, the reduction in hepatic burden in *Ifnar1^−/−^* mice was greatest on day 14 p.i. compared with controls, with around 3-fold fewer parasites found in the livers of these mice. Furthermore, resolution of hepatic infection was faster in *Ifnar1^−/−^* mice. A chronic infection was established in the spleens of WT mice, as expected (Engwerda and Kaye, 2000). However, control of parasite growth in the spleens of *Ifnar1^−/−^* mice was significantly better and, by day 56 p.i., was around 20-fold lower than in WT mice. The latter observation was associated with a significant reduction in hepatosplenomegaly at later time points in mice lacking type I IFN signaling pathways (Figure 3B). Collectively, these data identify a deleterious role of type I IFNs in control of *L. donovani* infection in C57BL/6 mice.

As a first step to try and understand how type I IFN signaling pathways suppressed anti-parasitic immunity, we measured serum IFNγ, tumor necrosis factor (TNF), and IL-10 levels over the course of *L. donovani* infection in *Ifnar1^−/−^* and WT control mice (Figure 3C). We focused on these cytokines because of their key roles in determining disease outcome in experimental VL (Murphy et al., 2001; Taylor and Murray, 1997; Tumang et al., 1994). Elevated levels of IFNγ and TNF were found in *Ifnar1^−/−^* mice on day 14 p.i. compared with WT controls, but the levels subsequently declined over the course of infection, and TNF levels were significantly lower in *Ifnar1^−/−^* mice on days 28 and 56 p.i. compared with WT controls. No differences in serum IL-10 levels were found throughout the course of infection. We also measured *Ifng, Tnf,* and *Il10* mRNA levels in the spleen and liver of *L. donovani*-infected *Ifnar1^−/−^* and WT mice on day 14 p.i. and found few differences in the spleen, but significantly increased *Ifng* and *Tnf* mRNA levels in *Ifnar1^−/−^* mice compared with WT mice (Figure S1). Little change in *Il10* mRNA was measured. In support of a role of type I IFN signaling in suppressing pro-inflammatory cytokine production, we stimulated splenocytes isolated from *Ifnar1^−/−^* and WT mice on day 14 p.i. with parasite antigen and found increased IFNγ and TNF but not IL-10 production in cells from *Ifnar1^−/−^* compared with cells from WT mice (Figure 3D). Thus, despite no significant increase in splenic *Ifng* and *Tnf* mRNA levels in *Ifnar1^−/−^* mice, splenocytes from these mice had a greater capacity for IFNγ and TNF production following antigen stimulation compared with WT splenocytes on day 14 p.i.

**Figure 4.**
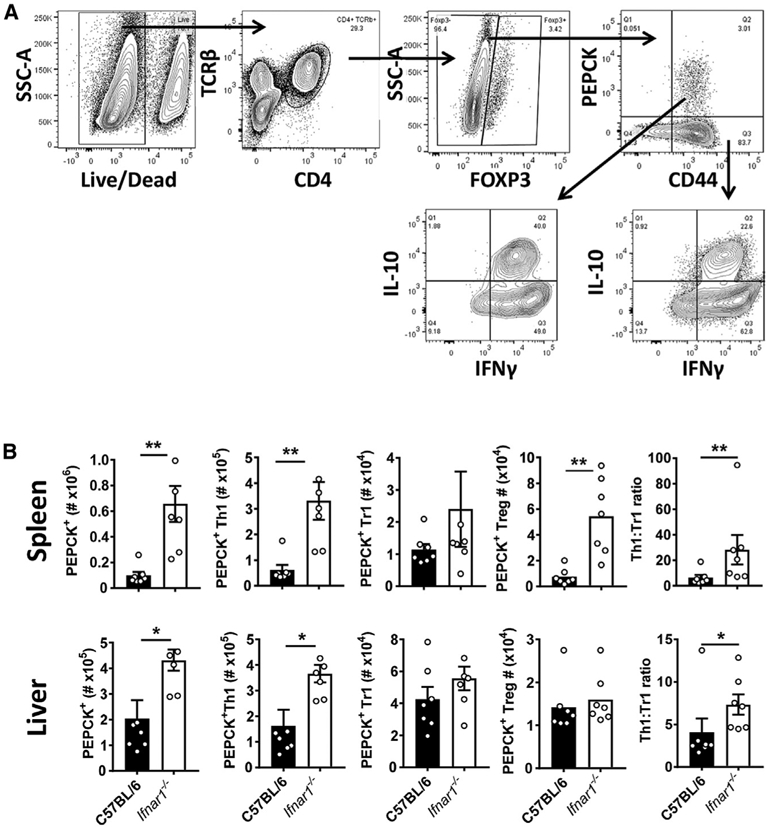
*Ifnar1*-Deficient Mice Have Improved Parasite-Specific CD4^+^ T Cell Responses (A) A *Leishmania*-specific MHCII-specific tetramer presenting the PEPCK peptide was used to measure parasite-specific CD4^+^ T cell responses using the gating strategy for liver mononuclear cells. (B) *Ifnar1^−/−^* (open columns) and control B6 WT (closed columns) mice were infected with *L. donovani* for 14 days prior to measuring PEPCK^+^ CD4^+^ T cells and Th1 cells, Tr1 cells, and Treg cells in the spleen and liver, as indicated. n = 7 mice per group. Experiments were conducted 3 times. Mean ± SEM; *p < 0.05 and **p < 0.01; significance assessed by Mann-Whitney test.

To test whether the above effects of type I IFN signaling were related to parasite-specific CD4^+^ T cell responses, we employed a recently developed *Leishmania*-specific MHC class II-specific tetramer presenting the phosphoenolpyruvate carboxykinase (PEPCK) peptide (Mou et al., 2015) to measure antigen-specific CD4^+^ T cell populations on day 14 p.i. (Figure 4A). Supporting our results above, we found increased numbers of antigen-specific CD4^+^ T cells and Th1 cells in the spleen and liver of *Ifnar1^−/−^* mice compared with WT mice (Figure 4B). Interestingly, we found no increase in the number of antigen-specific IL-10-producing Th1 (Tr1) cells, a major regulatory CD4^+^ T cell subset in VL (Montes de Oca et al., 2016a; Nylén and Sacks, 2007; Stäger et al., 2006) (Figure 4B). However, the ratio of antigen-specific Th1:Tr1 cells was always significantly greater in *Ifnar1^−/−^* mice compared with WT mice (Figure 4B). Similar results were also found when we examined the polyclonal CD4^+^ T cell response in the same mice (Figure S2). The frequency of antigen-specific and polyclonal Th1 cells was not increased in *Ifnar1^−/−^* mice compared with WT mice (Figure S2). However, Tr1 cell frequencies were always reduced, again making the ratio of Th1:Tr1 cells significantly greater in *Ifnar1^−/−^* mice compared with WT mice (Figure S2). We also found decreased frequencies of antigen-specific and polyclonal FoxP3^+^ CD4^+^ regulatory T (Treg) cells in the livers of *Ifnar1^−/−^* mice compared with WT mice, but changes in the spleen were less consistent (Figure S2). We further examined CD8^+^ T cells, and despite increased numbers in the spleen and liver of *Ifnar1^−/−^* mice compared with WT mice, their frequency was diminished, as was the frequency of IFNγ-producing CD8^+^ T cells (Figure S3). Thus, type I IFNs produced following *L. donovani* infection of C57BL/6 mice suppressed development of parasite-specific Th1 cell responses and/or promoted Tr1 cell development associated with impaired control of parasite growth.

### Improved Control of Parasite Growth in the Absence of Type I IFNs Required CD4^+^ T Cells and IFNγ

**Figure 5.**
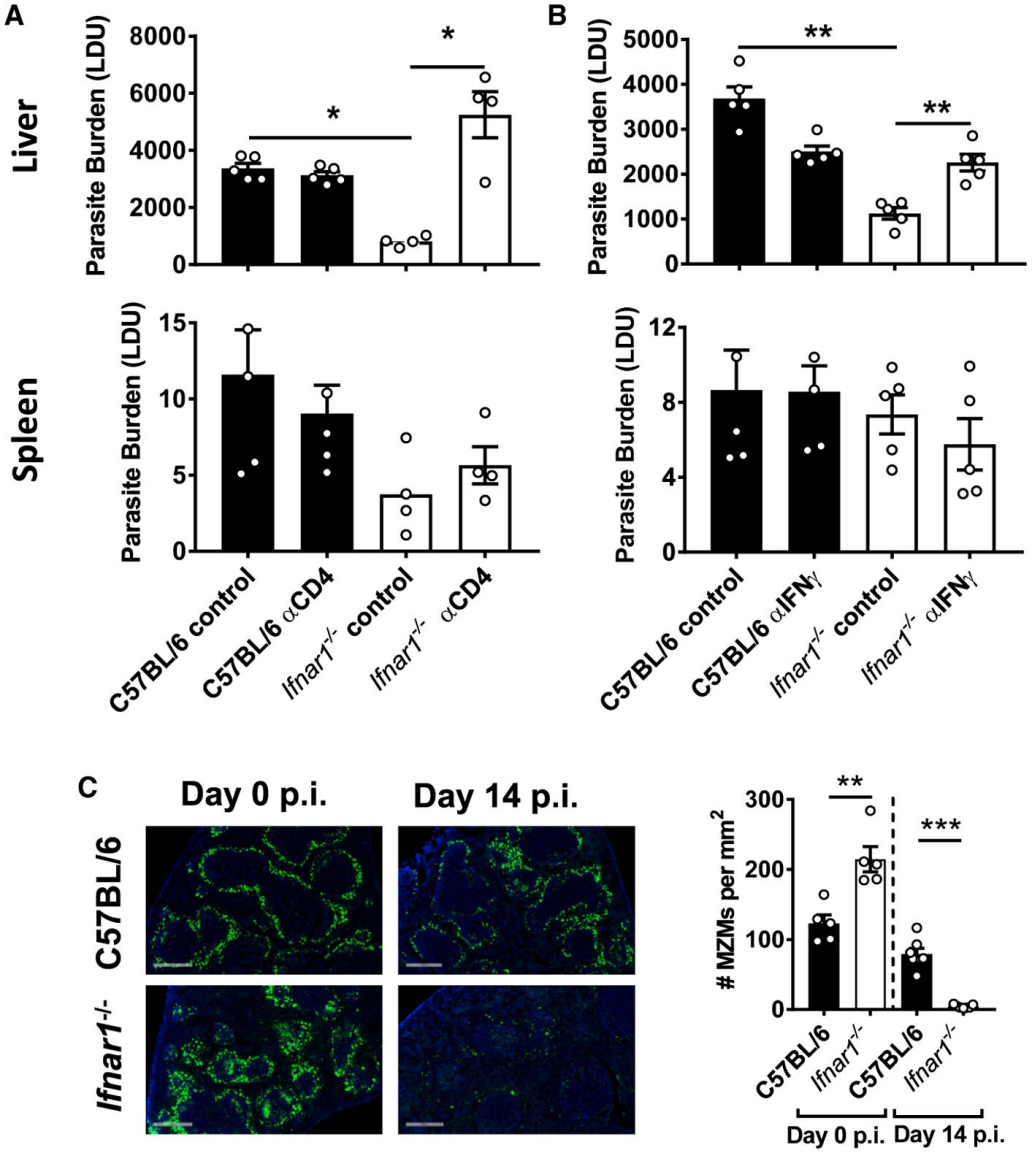
Improved Control of Parasite Growth in the Absence of Type I IFNs Required CD4^+^ T Cells and IFNγ (A and B) *Ifnar1^−/−^* (open columns) and control B6 WT (closed columns) mice were infected with *L. donovani* and treated with a depleting anti-CD4 mAb (A) or a blocking anti-IFNγ mAb (B). Parasite burdens were measured in the liver and spleen, as indicated, on day 14 post-infection (p.i.) and compared with mice treated with control antibodies. (C) The number of MZ macrophages (MZMs) per square millimeter of spleen tissue was determined on day 14 p.i., as indicated. Representative images show nucleated cells (blue, DAPI) and MZMs (indicated by uptake of fluorescein isothiocyanate (FITC)-dextran, green) (objective, 20×; scale bars, 500 μm). n = 4–7 mice per group. Experiments were conducted 2–3 times. Mean ± SEM; *p < 0.05, **p < 0.01, and ***p < 0.001; significance assessed by one-way ANOVA or Mann-Whitney test, as appropriate.

The above results indicate important roles of CD4^+^ T cells and IFNγ in improved control of parasite growth in *Ifnar1^−/−^* mice. To test whether this was the case, we infected *Ifnar1^−/−^* and WT mice with *L. donovani* and either depleted CD4^+^ T cells or blocked IFNγ activity. With both treatments, we found increased hepatic parasite growth in *Ifnar1^−/−^* mice relative to control animals (Figure 5), demonstrating a critical role of CD4^+^ T cells (Figure 5A) and IFNγ (Figure 5B) in improved control of parasite growth in the absence of type I IFN signals. No differences were found in hepatic parasite growth in WT mice, regardless of treatment, in agreement with previous studies showing that the differences from such treatment do not become apparent until 4 weeks after infection (Bunn et al., 2014; Engwerda et al., 1998; Murray et al., 1992). In addition, no effect on splenic parasite growth was observed, possibly reflecting the relatively slow and heterogeneous parasite growth in this tissue site (Engwerda and Kaye, 2000; Kaye et al., 2004).

One potential consequence of increased inflammation following *L. donovani* infection is accelerated breakdown of splenic architecture (Engwerda et al., 2002; Montes de Oca et al., 2016a), and, in fact, this was the case in *Ifnar1^−/−^* mice on day 14 p.i. compared with WT control mice (Figure 5C). In the absence of type I IFN signaling, there was a dramatic loss of marginal zone (MZ) macrophages from the spleen on day 14 p.i., a process mediated by IFNγ and TNF production (Engwerda et al., 2002; Montes de Oca et al., 2016a). Interestingly, this was despite *Ifnar1^−/−^* mice starting with a greater concentration of MZ macrophages in splenic tissue prior to infection compared with control WT mice (Figure 5C). Thus, the improved control of parasite growth in the absence of type I IFN signaling was dependent on CD4^+^ T cells and IFNγ but came at the cost of more rapid development of tissue pathology associated with the acute inflammation generated in the first 14 days of infection.

### Type I IFNs Act on DCs to Suppress Development of Antigen-Specific CD4^+^ T Cells

**Figure 6.**
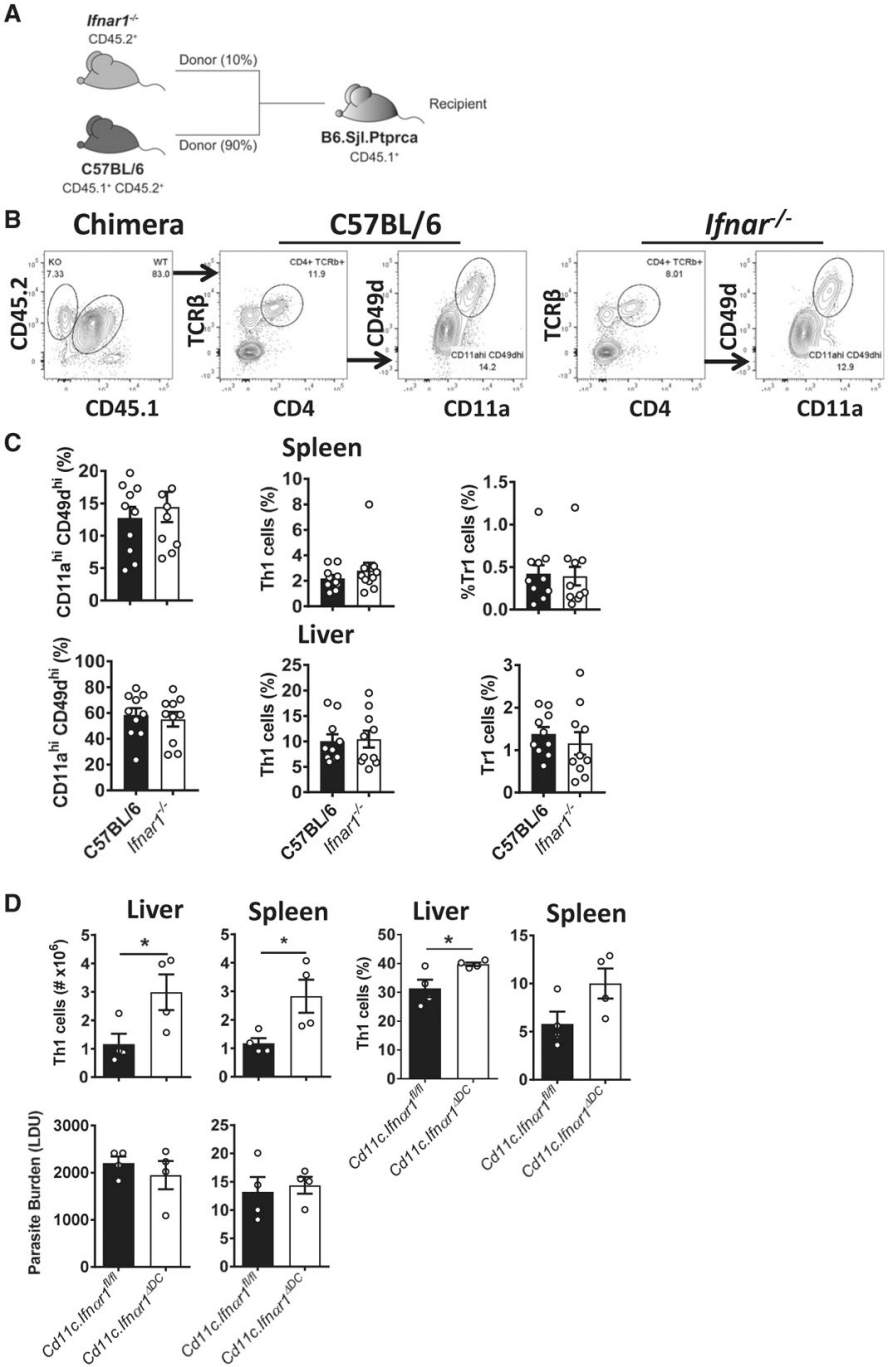
Type I IFNs Act on DCs to Suppress Development of Antigen-Specific CD4^+^ T Cells (A) A 90:10 mix of B6.CD45.1/CD45.2 and *Ifnar1^−/−^* (CD45.2) BM cells was engrafted into lethally irradiated CD45.1 recipients. (B) Following 10–12 weeks of engraftment, chimeric mice were infected with *L. donovani*, and 14 days later, spleen and liver mononuclear cells were isolated to assess the activation status of control B6 (CD45.1/CD45.2) and *Ifnar1^−/−^* (CD45.2) CD4^+^ T cells from the same mouse using the gating strategy shown. (C) The frequency of splenic and hepatic CD4^+^ T cells recently activated by parasite antigen (CD11a^hi^/CD49d^hi^), Th1 cells, and Tr1 cells (gated as shown in Figure S2) was measured by flow cytometry, as indicated. (D) *Ifnar1^ΔDC^* mice generated by crossing *Cd11c-Cre* mice with *Ifnar1*-floxed animals were infected with *L. donovani*, and 14 days later, Th1 cell number and frequency as well as parasite burden in the spleen and liver were measured, as indicated. n = 4–10 mice per group. Experiments were conducted 3 times. Mean ± SEM (C and D), *p < 0.05; significance assessed by Mann-Whitney test.

To establish whether type I IFNs acted directly on CD4^+^ T cells to mediate their suppressive effects during *L. donovani* infection, we generated mixed bone marrow (BM) chimeric mice by lethally irradiating congenic (CD45.1) B6 WT mice and engrafting them with a 90:10 mix of BM from B6 WT (CD45.1 and CD45.2) and *Ifnar1^−/−^* (CD45.2) mice (Figure 6A). After 12 weeks of engraftment, chimeric mice were infected with *L. donovani*, and parasite-specific CD4^+^ T cells responses were measured 14 days later (Figure 6B). We found no change in the frequency of *Ifnar1^−/−^* (CD45.2) CD4^+^ T cells recently exposed to antigen (CD11a^hi^ CD49d^hi^; Butler et al., 2011), Th1 cells, or Tr1 cells compared with corresponding cell populations of control C57BL/6 WT (CD45.1 and CD45.2) origin (Figure 6C), indicating that type I IFNs were not signaling directly to CD4^+^ T cells to enhance their pro-inflammatory activity.

We showed previously, in a mouse malaria model, that DCs are a primary target for mediating the suppressive activity of type I IFNs on CD4^+^ T cell responses (Haque et al., 2014). To test whether this was also the case in experimental VL, we first assessed DC activation in the spleen 5 h after infection. This time point was chosen because we and other have shown previously that DCs produce peak amounts of IL-12 in the spleen at this time after infection (Engwerda et al., 1998; Maroof and Kaye, 2008). However, we found limited changes in DC number and frequency as well as activation status (assessed by CD86 expression) in *Ifnar1^−/−^* mice compared with WT mice (Figures S4A and S4B). In addition, although IL-12 and IL-10 mRNA levels were elevated in *Ifnar1^−/−^* DCs compared with WT DCs, this only reached statistical significance for Il10 mRNA levels (Figure S4C). We also examined DC and monocyte number, frequency, and activation status in the spleen and liver 14 days after *L. donovani* infection, when differences in Th1 cell responses were observed. We found significantly increased DC and monocyte numbers in both organs in *Ifnar1^−/−^* mice compared with WT mice (Figures S5A-S5C). Furthermore, the number of activated monocytes, as indicated by MHC class II expression, was also increased in these mice compared with controls (Figure S5C). However, these differences were no longer apparent when frequencies of cells were examined, presumably because of the more rapid expansion of lymphocyte subsets in *L. donovani*-infected *Ifnar1^−/−^* mice compared with WT mice.

Given the above change in DC numbers, we next tested whether type I IFNs were acting directly on DCs by infecting *Ifnar1^ΔDC^* mice generated by crossing *Cd11c-Cre* mice with *Ifnar1*-floxed mice (Haque et al., 2014). Indeed, increased Th1 cell responses were found in *L. donovani*-infected mice with *Ifnar1*-deficient DCs relative to littermate controls (Figure 6D). However, there was no improvement in parasite control in these mice (Figure 6D), possibly indicating involvement of other *Ifnar1*-expressing cells in control of parasite growth. We also generated mice with *Ifnar1*-deficient myeloid cells by crossing *Lysm-Cre* and *Ifnar1*-floxed mice but could only obtain *Ifnar1* deletion in 40%–60% of monocytes and macrophages, consistent with previous reports (Abram et al., 2014; Bunn et al., 2018). Hence, although these mice had no change in anti-parasitic immunity relative to controls following *L. donovani* infection (data not shown), the results could not be interpreted because of inefficient *Ifnar1* deletion. However, to establish whether monocytes might be a target for type I IFNs and help explain the phenotype of in *Ifnar1^−/−^* mice during experimental VL, we examined the response of *Ccr2*-deficient mice to *L. donovani* infection. As expected, monocyte recruitment into inflamed tissue, such as the liver, during VL was impeded in these animals (Figures S6A and S6B). Associated with defective hepatic monocyte recruitment in *Ccr2^−/−^* mice were reduced numbers of recruited CD4^+^ T cells and frequency of Th1 cells compared with WT control mice and increased parasite burden (Figure S6B). These findings indicate a role for inflammatory monocytes in the recruitment and/or expansion of Th1 cells in the liver of *L. donovani*-infected mice. We found no intrinsic defect in the ability of *Ifnar1^−/−^* peritoneal macrophages to control *L. donovani* growth, compared with WT macrophages (Figures S6C and S6D), suggesting that type I IFNs did not directly impede the ability of host macrophages to control parasite growth. Thus, type I IFNs act on DCs to suppress priming and/or expansion of Th1 cells during *L. donovani* infection, but other type I IFN targets, such as monocytes, may also contribute to the negative effects of type I IFNs on the development anti-parasitic immune responses following *L. donovani* infection.

### Blockade of Type I IFN Signaling with Ruxolitinib Acts with Ambisome to Improve Anti-parasitic Immunity

**Figure 7.**
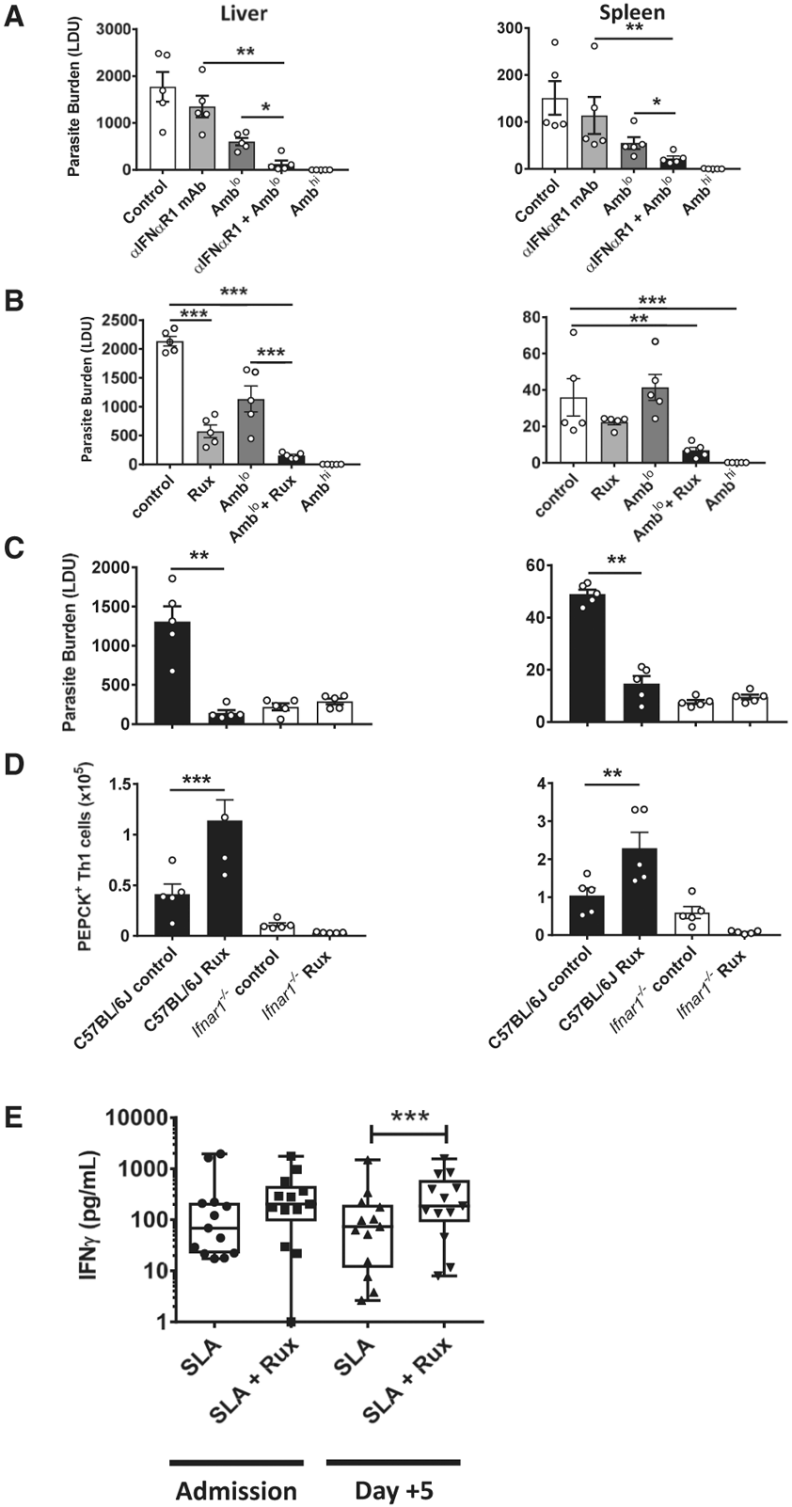
Type I IFN Signaling Blockade Acts Synergistically with Ambisome to Improve Anti-parasitic Immunity (A–D) B6 WT mice were infected with *L. donovani* and, 14 days later, treated with either vehicle control, a single suboptimal dose of Ambisome (Amb^lo^, 1 mg/kg) with or without 0.1 mg anti-IFNαR1 mAb every 3 days for 14 days (A), or with or without 60 mg/kg ruxolitinib (Rux) twice a day for 5 days (B). Parasite burdens in the liver and spleen were measured on day 28 p.i., as indicated, and a full dose of Ambisome (Amb^hi^, 10 mg/kg) was used as a positive control for drug efficacy. *Ifnar1^−/−^* (open columns) and control B6 WT (closed columns) mice were infected with *L. donovani* for 14 days and then treated with 60 mg/kg Rux twice a day for 5 days. Parasite burden (C) and number of parasite-specific CD4^+^ T cells (gated as in Figure 4A) (D) were measured on day 28 p.i., as indicated. n = 4–7 mice per group. Experiments were conducted twice. Mean ± SEM; *p < 0.05, **p < 0.01, and ***p < 0.001; significance assessed by one-way ANOVA or Mann-Whitney test, as appropriate. (E) Antigen-specific IFN-γ production was measured in whole-blood cells from VL patients upon admission to the clinic and, 3 days after Ambisome treatment (n = 13), cultured for 24 h with SLA with vehicle control or Rux (1 μM), as indicated. Median + minimum and maximum; *p < 0.05; significance assessed by Wilcoxon matched-pairs signed-rank test.

To investigate the therapeutic potential of blocking type I IFN signaling, we first tested an anti-IFNαR1 monoclonal antibody (mAb) (Sheehan et al., 2006) in an established infection (Figure 7A). Given that host-directed therapies aimed at improving control of infectious diseases are likely to be used in association with conventional anti-parasitic drugs, we tested anti-IFNαR1 mAb treatment with and without a suboptimal dose of Ambisome, the first-line drug for treating VL in India (Sundar et al., 2010). A suboptimal dose of the drug was employed to better identify additive or synergistic effects of type I IFN signaling blockade. C57BL/6 WT mice were infected with *L. donovani* for 14 days and then treated with a single dose of Ambisome and/or the anti-IFNαR1 mAb. Mice that received an optimal dose of Ambisome efficiently controlled parasites, whereas the suboptimal dose of Ambisome had approximately 50% better control of parasite growth on day 28 p.i. than mice treated with the vehicle control (Figure 7A). No significant reduction in parasite growth was found in mice treated with anti-IFNαR1 mAb alone. However, mice that received both sub-optimal Ambisome and the anti-IFNαR1 mAb had significantly better parasite control in the liver and spleen compared with mice receiving monotherapy, indicating an additive effect of type I IFN blockade and anti-parasitic drug treatment.

We next turned our attention to identifying a small-molecule inhibitor of type I IFN signaling that might be more suitable for treating a neglected tropical disease in a field setting. Ruxolitinib (Jakafi or Jakavi) is a licensed, orally administered JAK1/JAK2 inhibitor used to treat neoplastic diseases, particularly myelofibrosis (Harrison et al., 2012; Verstovsek et al., 2012). Recently, this drug was used with some success to treat children with a type I interferonopathy (Frémond et al., 2016) and dermatomyositis patients with type I IFN-mediated disruption of vascular endothelial networks (Ladislau et al., 2018). Therefore, we repeated the above therapeutic protocol but replaced the anti-IFNαR1 mAb with ruxolitinib. Remarkably, mice treated with ruxolitinib alone had significantly improved control of parasite growth in the liver and in both liver and spleen when combined with the suboptimal dose of Ambisome compared with vehicle control-treated mice (Figure 7B). Thus, ruxolitinib had a similar but more dramatic anti-parasitic effect as the anti-IFNαR1 mAb, suggesting that it targeted the same type I IFN signaling pathway.

Given that JAK1 and JAK2 participate in a number of cytokine signaling pathways, we next examined whether ruxolitinib was indeed targeting type I IFN signaling in *L. donovani*-infected mice by treating B6 WT mice with ruxolitinib and comparing their response to *Ifnar1^−/−^* mice (Figure 7C). In contrast to improved control of parasite growth in B6 WT mice treated with ruxolitinib, there was no improvement in the liver or spleen of ruxolitinib-treated *Ifnar1^−/−^* mice compared with control-treated WT mice. This was associated with an approximately 2-fold increase in the number of parasite-specific Th1 cells in the livers and spleens of ruxolitinib-treated B6 WT mice but no increase in ruxolitinib-treated *Ifnar1^−/−^* mice relative to vehicle control-treated WT mice (Figure 7D). Of note, there was no increase in antigen-specific Th1 cell numbers in control *Ifnar1^−/−^* mice relative to control B6 WT mice at this day 28 p.i. time point, possibly reflecting a rapid contraction of inflammatory responses in the former strain after day 14 p.i. Similar results were also found when we measured the polyclonal Th1 cell responses in the same mice (Figure S7). Hence, the anti-parasitic effect of ruxolitinib treatment was not observed in the absence of type I IFN signaling.

### Increased Effect of Type I IFN Blockade on Anti-parasitic Immune Responses after Treatment of VL Patients with Ambisome

Finally, we examined whether similar improvements in anti-parasitic immunity in humans were generated by blocking type I IFN signaling. Treatment of VL patients with anti-parasitic drugs such as Ambisome kills parasites, resulting in increased availability of parasite material that can stimulate type I IFN production. Given our results above, we hypothesized that this would limit the development of anti-parasitic immunity. To test this, we again employed a WBA using VL patient blood samples at the time of admission to the hospital (before drug treatment) and 5 days after drug treatment. We again observed increased parasite-specific IFNγ production by blood cells following addition of ruxolitinib, relative to controls, both pre- and post-drug treatment (Figure 7E). However, this increase only reached statistical significance 5 days after drug treatment. Thus, type I IFN-mediated suppression of parasite-specific IFNγ production in VL patient blood cells was overcome by ruxolitinib, and this effect was greatest after VL patients had been treated with Ambisome.

## DISCUSSION

In this study, we identified type I IFNs as major negative upstream regulators of CD4^+^ T cell anti-parasitic activity in VL. This cytokine family did not act directly on CD4^+^ T cells but, instead, influenced accessory cell functions. Importantly, we demonstrated the therapeutic potential of targeting this pathway for clinical advantage in a pre-clinical disease model as well as with clinical samples, using both mAbs and a small-molecule inhibitor that blocked type I IFN signaling. The latter JAK1/2 inhibitor is an available, licensed, orally administered drug.

The role of type I IFNs in leishmaniasis varies depending on the disease and causative species (Silva-Barrios and Stäger, 2017). Early studies using poly(I:C) to stimulate type I IFNs in mice infected with *L. donovani* found that treatment prior to infection had a positive effect on disease outcome, whereas treatment during infection suppressed anti-parasitic immunity (Herman and Baron, 1970), consistent with results in the current study. Similarly, STAT1 plays an important role in type I IFN signaling (Bromberg and Darnell, 2000), and *Stat1*-deficient mice were relatively resistant to *L. donovani* infection compared with WT controls (Rosas et al., 2006). Type I IFN signaling to B cells was also found to suppress anti-parasitic immune responses in mice infected with *L. donovani* (Silva-Barrios et al., 2016), but not as dramatically as reported here in infected *Ifnar1^−/−^* mice. The less striking effect in mice with *Ifnar1*-deficient B cells was akin to the partial effect in *L. donovani*-infected mice with *Ifnar1*-deficient DCs, where, despite increased Th1 cell responses, there was no effect on parasite burdens. Data from experimental cutaneous leishmaniasis caused by *L. guyanensis* showed that type I IFN production stimulated by a parasite endogenous virus affected infected macrophages, causing increased parasite growth and dissemination (Rossi et al., 2017), identifying this host cell population as a potential target for the immunosuppressive effects of type I IFNs during leishmaniasis, along with DCs, which we have shown previously to be targeted by type I IFNs in an experimental malaria model (Haque et al., 2014). Indeed, our results from *Ccr2*-deficient mice, where there was a defect in inflammatory monocyte recruitment to the liver during *L. donovani* infection, indicate an important role of monocytes in recruitment and/or maintenance of hepatic Th1 cell responses. However, whether monocyte migration or development into antigen-presenting cells is affected by type I IFNs is currently unknown. Together, these results suggest that type I IFN signaling affects the functions of multiple immune cell populations at different times during infection to mediate immunoregulatory effects during VL.

Type I IFNs are pleiotropic cytokines with critical roles in controlling many viral infections (Fensterl and Sen, 2009). However, they also have detrimental effects on immune responses to many bacteria and parasites (Mayer-Barber and Yan, 2017; Sebina and Haque, 2018; Silva-Barrios and Stäger, 2017). This has been extensively studied in mouse models of tuberculosis (Donovan et al., 2017; Moreira-Teixeira et al., 2018), where both IL-10-dependent and -independent mechanisms have been identified (Mayer-Barber et al., 2011; McNab et al., 2014; Moreira-Teixeira et al., 2017). Type I IFN-mediated IL-10 production by CD4^+^ T cells suppressed Th1 cell responses in mice infected with *Mycobacterium tuberculosis* (Moreira-Teixeira et al., 2017). CD4^+^ T cell-derived IL-10 plays a similar role in mice infected with *L. donovani* (Bunn et al., 2018), and our results indicate that type I IFNs promote these responses at the expense of Th1 cell responses, as indicated in the increased ratio of Th1:Tr1 cells in *Ifnar1*-deficient *L. donovani*-infected mice. We also found reduced frequencies of Treg cells in *Ifnar1^−/−^* mice compared with WT controls. However, we recently showed that depletion of these cells had little effect on disease outcome in experimental VL, suggesting that this cellular change was likely to explain the improved control of parasite growth in the absence of type I IFN signaling. Instead, our results support a model where type I IFNs suppress Th1 cell responses in mice and humans infected with *L. donovani* through pathways dependent on type I IFN signaling through accessory cell populations. Further work is needed to fully characterize type I IFN-mediated immune modulation during VL. In particular, it will be important to establish the relationship between type I IFN and IL-1 cytokine pathways and whether they antagonize each other in a similar manner as during *M. tuberculosis* infection (Mayer-Barber and Yan, 2017). This may be important because inflammasome-mediated IL-1β production is needed to generate reactive nitrogen intermediates for parasite killing in *L. infantum chagasi*-infected mice (Lima-Junior et al., 2013). Of note, although CD4^+^ T cells might not be expected to be a major source of IL-1β, mRNA encoding this cytokine, along with that encoded by IL1R, was among the top 50 most downregulated genes in CD4^+^ T cells from VL patients compared with the same cells from ECs.

The current first-line treatment for VL in the Indian subcontinent is a single dose of Ambisome, whereas in East Africa, daily sodium stibocluconate in combination with intramuscular paromycin over 17 days is used (Burza et al., 2018). Problems with the former monotherapy include cost, toxicity, and risk of parasites developing drug resistance, whereas the latter combination treatment regime has the disadvantages of being intensive, with a relatively high risk of disease relapse as well as development of Post-kala-azar dermal leishmaniasis (PKDL) (Burza et al., 2018; Croft et al., 2006; Singh et al., 2016), a disease complication characterized by the presence of hypopigmented macular erythematous maculopapular rashes on the skin (Zijlstra et al., 2003). Therefore, complementing drug treatment with host-directed strategies aimed at improving anti-parasitic immunity would be of value (Kumar et al., 2017; Singh and Sundar, 2014), especially in the context of increased efforts to eliminate VL in the Indian subcontinent, where maintenance of long-term immunity within endemic communities will be important until parasite transmission cycles are disrupted. Hence, our finding that type I IFN blockade can be employed with anti-parasitic drugs to improve anti-parasitic immunity highlights one such strategy. Importantly, by identifying the small molecule JAK1/2 inhibitor ruxolitinib as an effective host-directed therapy, we have a drug that can be readily tested, given its oral route of administration and known safety profile. As well as being used to treat neoplastic diseases (Harrison et al., 2012; Verstovsek et al., 2012), this drug is used successfully to treat children with a type I interferonopathy associated with gain-of-function mutations in *TMEM173* (encoding stimulator of interferon genes [STING]), responsible for high childhood morbidity and mortality in affected individuals (Frémond et al., 2016). In addition, ruxolitinib reduces serum type I IFN levels and IFN-inducible gene scores in dermatomyositis patients (Ladislau et al., 2018). Thus, there is a growing body of evidence that this drug blocks type I IFN signaling in humans in a range of diseases.

One potential confounding factor of using this drug is that, by inhibiting JAKs, pro-inflammatory cytokine signaling pathways that are needed to control latent infections will also be suppressed. Indeed, there have been reports of myelofibrosis patients being treated with ruxolitinib presenting with reactivation of latent tuberculosis (Colomba et al., 2012; Hopman et al., 2014; Palandri et al., 2015). However, these patients are generally on long-term daily treatment with the drug, whereas we propose using the drug transiently at the time of anti-parasitic drug treatment. In fact, our data indicate that, when used in this way, the Th1 cell responses needed to control parasite growth were not only maintained but enhanced. A similar effect was observed in mice treated with a virus expressing IFNβ to treat plasmacytoma; ruxolitinib was able to rescue mice from lethal IFNβ toxicity without affecting anti-tumor immunity (Peng et al., 2017). Thus, transient blockade of type I IFN signaling during drug treatment when parasite products are present to drive type I IFN production should be considered an option to direct host immune responses toward protective anti-parasitic immunity.

In conclusion, we identified type I IFNs as critical but indirect regulators of anti-parasitic CD4^+^ T cells responses in VL caused by *L. donovani*. They reduce the number and frequency of Th1 cells relative to Tr1 cells. Additionally, we showed that targeting type I IFN signaling during anti-parasitic drug treatment can improve Th1 cell-mediated immunity. Together, these findings identify an approach that can be employed to help control and eliminate an major parasitic disease with limited treatment options and no effective vaccine.

## STAR★METHODS

### LEAD CONTACT AND MATERIALS AVAILABILITY

Further information and requests for resources and reagents should be directed to and will be fulfilled by the Lead Contact, Christian Engwerda (christian.engwerda@qimrberghofer.edu.au). This study did not generate any new unique reagents.

### EXPERIMENTAL MODEL AND SUBJECT DETAILS

#### Human subjects

Blood samples were collected from 55 symptomatic VL patients at the Kala-Azar Medical Research Centre (Muzaffarpur, Bihar, India.). Diagnosis was performed by detection of anti-rK39 antibodies in the serum and/or amastigotes in splenic biopsies. Clinical data for all subjects enrolled in the study is presented in Table 1. 5 mL of heparinised venous blood was collected per patient on admission to hospital (D-0) and processed immediately. Patients were treated with a single dose of AmBisome (10 mg/kg; Gilead Sciences, Inc., Foster City CA, U.S.A.) administered intravenously (i.v.). Blood was then collected from VL patients on discharge from hospital (D-Dis). Blood was also collected from 18 endemic controls (EC). Blood continued to be collected from VL patients and ECs over a period of 18 months. The study was approved by the Institute of Medical Sciences, Banaras Hindu University Ethics Committee and all subjects provided written informed consent. All patients were HIV negative and above twelve years of age.

#### Mice

Female mice between 6-12 weeks of age were used for all experiments unless otherwise stated. All mice were age-matched, group-housed with a maximum of 6 mice per cage and maintained under pathogen-free conditions at the QIMR Berghofer Medical Research Institute Animal Facility (Herston, QLD, Australia). B6.Cg-Tg(Itgax-cre)1-1Reiz/J (B6.*CD11c-cre*; Caton et al., 2007; RRID: IMSR_JAX:008068) mice were crossed to B6(Cg)-*Ifnar1*^*tm1.1Ees*^/J (*B6.Ifnar1*-floxed; Prigge et al., 2015; RRID: IMSR_JAX:028256) to generate mice that had close to 100% deletion of floxed *Ifnar1* genes in conventional CD11c^hi^ DC populations (B6.*Ifnar1^ΔDC^*; Haque et al., 2014). B6.129P2-*Lyz2^tm1(cre)lfo^*/J (B6.*Lysm-Cre*; Clausen et al., 1999; RRID: IMSR_JAX:004781) mice were also crossed to B6.*Ifnar1*-floxed mice to generate mice with *Ifnar1*-deficient myeloid cells only obtaining *Ifnar1* deletion in 40%–60% of monocytes and macrophages. Inbred female C57BL/6JArc (B6; RRID: IMSR_ARC:B6) mice and congenic female B6.SJL-Ptprc^a^Pepc^b^/BoyJArc (*Ptprc*; RRID: IMSR_JAX:002014) mice were both sourced from the Animal Resource Centre (ARC; Canning Vale, WA, Australia). All other mice were bred in-house including B6.(Cg)-*Ifnar1^tm1.2Ees^*/J (B6.*Ifnar1^−/−^*; Prigge et al., 2015; RRID: IMSR_JAX:028288), B6.129S4-*^Ccr2tm1lfc^*/J (B6.*Ccr2^−/−^*; Boring et al., 1997; RRID: IMSR_JAX:004999) and (B6.129S7-*Rag1^tm1Mom^*/J (B6.*Rag1^−/−^*; Mombaerts et al., 1992; RRID: IMSR_JAX:002216). Where the same strain of mice was spread across multiple experimental groups, littermates were distributed randomly into groups. All animal procedures were conducted with the approval of the QIMR Berghofer Medical Research Institute Animal Ethics Committee under the animal ethics number A02-634M and in accordance with the “Australian Code of Practice for the Care and Use of Animals for Scientific Purposes” (Australian National Health and Medical Research Council (NHMRC)).

#### L. donovani strains

*L. donovani* stationary-phase promastigotes used to prepare soluble *Leishmania* antigen (SLA) for human PBMC *ex vivo* whole blood assay were cultured from *L. donovani* amastigotes isolated from VL patient splenic aspirates (Kala-Azar Medical Research Centre, Muzaffarpur, Bihar, India). *L. donovani* amastigotes (LV9; MHOM/ET/67/HU3) used for *in vivo* and *in vitro* murine infections was originally isolated from a patient in Ethiopia in 1967 (Bradley and Kirkley, 1977) and maintained by passage in B6.*Rag1^−/−^* mice.

### METHOD DETAILS

#### Human PBMC isolation and RNA extraction

Blood was collected from VL patients and ECs into BD Vacutainer® Lithium Heparin^N^ (LH) 170 I.U. Plus Blood Collection Tubes (BD Biosciences). Blood was layered over Lymphoprep (StemCell Technologies, Inc.) to isolate PBMCs. CD4^+^ T cells were isolated from PBMCs by magnetic-activated cell sorting (MACS) using MS Columns and anti-human CD4 MicroBeads (both by Miltenyi Biotec, Bergisch Gladback, Germany) into RNA*later* (Invitrogen, Carlsbad, CA). Cells were homogenized using the QIAshredder and RNA isolated using the RNeasy Mini Kit (both by QIAGEN, Hilden, Germany) according to manufacturer’s instructions. DNA digestion was performed using either the RNAase-free DNase set or DNase Max kit (both by QIAGEN). RNA was quantified using the Qubit RNA HS Assay kit on a Qubit 4 Fluorometer (Thermo Fisher, Waltham, MA). The quality of RNA was determined using the RNA 6000 Nano kit, run on a 2100 Bioanalyzer (both by Agilent Technologies, Santa Clara, CA) according to the manufacturer’s instructions where an RNA Integrity Number (RIN) value of above 8 was optimal.

#### RNA-sequencing

Libraries were prepared using the TruSeq Stranded mRNA Library Prep Kit, High Throughput (Illumina, San Diego, CA) with Superscript III Reverse Transcriptase (Life Technologies, Carlsbad, CA) and Agencourt AMPure XP beads (Beckman Coulter, Brea, CA). Libraries prepared were quantified using the Qubit DNA HS Assay kit (Thermo Fisher) and quality was assessed using the DNA 100 kit (Agilent Technologies), run on a 2100 Bioanalyzer. 75bp, paired-end RNA-sequencing was performed on the NextSeq 550 using the NextSeq 500/550 High Output Kit v2 (150 cycles) (Illumina). Each flow cell contained 12 libraries.

#### Differential expression and pathway analysis

Adaptor sequences were trimmed from fastq files using Cutadapt (Martin, 2011) (version 1.11). Sequences were aligned to the GRCh37 assembly with the gene, transcript, and exon features of Ensembl (release 89) gene model, using STAR (Dobin et al., 2013) (version 2.5.2a). Quality control metrics were computed using RNA-SeQC (DeLuca et al., 2012) (version 1.1.8). Reads were then quantified using RSEM (Li and Dewey, 2011) (version 1.2.30). Normalization and differential gene expression analysis was performed using the edgeR package (Robinson et al., 2010). Ingenuity Pathway Analysis (IPA; winter 2018 release; QIAGEN) was used for pathways analysis.

#### Human PBMC RT-qPCR

PBMCs were isolated and RNA prepared as described above. Enriched cell populations from PBMCs were prepared using a sequential cell selection protocol previously described (Ansari et al., 2011; Nylén et al., 2007). Briefly, cell subsets were enriched by sequential MACS positive selection of CD19^+^ (B cells), followed by CD14^+^ (monocytes and macrophages) and CD1c^+^ (DCs). Selections were carried out using MACS beads and Magnetic LS columns (Miltenyi Biotec) according to the manufacturer’s instructions. cDNA synthesis was performed using a High-Capacity cDNA Archive kit, following the manufacturer’s instructions (Thermo Fisher). Real-time qPCR was performed using TaqMan based chemistries with FAM MGB-labeled primer/probes to measure mRNA expression, while VIC-MGB labeled 18S rRNA was used as an endogenous control, as previously described (Ansari et al., 2011), using an ABI-Prism 7500 (Thermo Fisher). Primers included sets designed to amplify human *IFNB1* (Hs01077958_s1), *IFNA1* (Hs00855471_g1), *IFNAR1* (Hs01066118_m1) and *IFNAR2* (Hs01022060_m1) (Thermo Fisher).

#### Preparation of soluble Leishmania antigen

Soluble *Leishmania* antigen (SLA) was prepared as previously described (Gidwani et al., 2011). Briefly, *L. donovani* amastigotes from clinical isolates (Kala-azar Medical Research Center, Muzaffapur, Bihar, India) were grown in Medium 199, Hanks’ Balanced Salts (M199; Thermo Fisher) until transformed into promastigotes, then cultured. 2 × 10^9^ stationary-phase promastigotes were harvested from culture and centrifuged at 3900 *g* for 20 minutes to obtain parasite pellet, which was washed three times with cold 1x PBS and resuspended in solution (10 mM Trizma® hydrochloride solution (TRIS-HCl; Sigma-Aldrich), 1 mM pH 8.0 ethylenediaminetetraacetic acid (EDTA; Amresco), 1.6 mM phenylmethanesulphonyl fluoride (PMSF; HiMedia, Mumbai, India), and 50 μg/ml N-acetyl-L-leucyl-L-leucyl-L-argininal (leupeptin; Amresco)) at a concentration of 2 × 10^9^ parasites/ml. The parasite suspension was sonicated 4-5 times for 15 s at 10 Hz and centrifuged at 27,000 *g* for 30 minutes at 4°C. The lipid layer was removed from the surface of supernatant and the remaining solution was ultracentrifuged at 100,000 g for 4 hours at 4°C. The supernatant was removed, dialysed against the PBS overnight and stored at −80°C until use. Protein was measured using a Micro BCA Protein Assay Kit (Thermo Fisher) as per manufacturer’s instructions.

#### *Ex vivo* whole blood assay

Whole blood assays were performed, as previously described (Ansari et al., 2011). Briefly, heparinised blood was collected from active VL patients. To remove background plasma cytokines, the plasma was removed and the whole blood was washed once with 1x PBS. The autologous plasma was then replaced with an equal volume of FBS. Whole blood cells were cultured in the absence or presence of SLA. To detect whether blockade of type I IFN signaling could enhance antigen specific immune responses, anti-IFN Receptor Chain 2 antibody (clone: MMHAR-2, final concentration 5 μg/ml; R&D System, Minneapolis, MN) or its mouse IgG2a isotype control (clone: 20102, final concentration 5 μg/ml, R&D Systems) were added to whole blood cell cultures. In some experiments, Ultra-LEAF Purified anti-human HLA-DR antibody (clone: L243, final concentration 20 μg/ml, BioLegend, San Diego, CA) or its Ultra-LEAF purified mouse IgG2a, κ isotype control antibody (clone: MOPC-173, final concentration 20 μg/ml, BioLegend) was added. In all experiments, a non-stimulated group was included, and although minimal cytokine production was detected in these samples, the levels detected were subtracted from corresponding antigen-stimulated samples. Whole blood cultures were kept at 37°C and 5% CO_2_ for 24 hours. Supernatants were collected and IFN-γ levels were measured using an ELISA kit (BioLegend, San Diego, CA), as per manufacturer’s instructions. To test the effect of drug treatment on responses to type I IFN signaling blockade in whole blood assays, a similar experiment was conducted on a separate set of whole blood samples, where heparinised blood was collected from active VL patients before the start of treatment and five days after a single-dose AmBisome treatment (Sundar et al., 2010), in the presence of 1 μM ruxolitinib (Rux; Santa Cruz Biotechnology) or an equal concentration of vehicle (Dimethylacetamide (DMAc); Sigma-Aldrich, St Louis, MO).

#### L. donovani *in vivo* infections in mice

*L. donovani* (LV9; MHOM/ET/67/HU3) were maintained by passage in C57BL/6 (B6) *Rag1^−/−^* mice. Chronically infected passage mice were euthanised and the spleen was excised into 5 mL of sterile Roswell Park Memorial Institute Medium 1640 (RPMI1640; GIBCO, Life Technologies, Carlsbad CA, USA) + 100 μg/ml penicillin–streptomycin (PS; GIBCO, Life Technologies); RPMI/PS) medium. The excised spleen was homogenized and the cell suspension was centrifuged in an Eppendorf Centrifuge 5810 R (Fisher Scientific, Thermo Fisher Scientific) at 115 *g* for 5 minutes at room temperature (RT), with brake off. The supernatant was transferred to a new tube, and the pellet discarded. The supernatant was centrifuged at 1960 *g* for 15 minutes at RT. The supernatant was discarded, and the pellet was incubated for 5 minutes in 1 mL of Red Blood Cell Lysing Buffer Hybri-Max (Sigma-Aldrich). Sterile RPMI/PS was added and centrifuged at 1960 *g* for 15 minutes at RT. Supernatant was again discarded and further RPMI/PS was added to pellet and centrifuged again at 1960 *g* for 15 minutes at RT. After discarding this final supernatant, the parasite pellet was re-suspended in approximately 1 mL sterile RPMI/PS. The parasites were repeatedly passed through a 26G x ½” needle on a 1 mL syringe (Terumo® Medical, Somerset NJ, USA) until a homogeneous suspension was achieved. Two microliters of the suspension were loaded onto a Thoma cell counting chamber (Weber Scientific International, West Sussex, UK) and parasites were counted in the 4 × 4 grid in triplicate. An average count was used to determine the number of parasites/ml using the following equation:

$$\frac{average}{16}\times 2\times 10^{7}=parasites∕ml$$

Parasites were diluted to a final concentration of 1 × 10^8^ parasites/ml in sterile RPMI/PS. Each experimental mouse received 2 × 10^7^ parasites in 200 μL via i.v. injection into the lateral tail vein.

#### Quantifying murine Leishmania parasite burden

Where indicated, parasite burden was quantified via histological assessment of Giemsa-stained (Diff-Quick; Lab Aids, Narrabeen, NSW, Australia) liver and spleen impression smears. The number of amastigotes per 1000 host nuclei were counted under x100 objective using a light microscope (Olympus CX31; Olympus Life Science, Shinjuku, Tokyo, Japan), multiplied by the organ weight (grams) and expressed as Leishman-Donovan Units (LDU).

#### Mice serum cytokine analysis

Where indicated, approximately 500 μL of murine blood was collected via cardiac puncture with 1.0 mL 27G BD Insulin Syringe (BD Medical) into a 1.5 mL Eppendorf tube. Blood collected was centrifuged at 14,000 *g* for 10 minutes in order to pellet cellular debris. Sera were then isolated, frozen and stored at −20°C prior to analysis. Serum IFNγ, TNF and IL-10 cytokine levels were detected using CBA Flex Sets (BD Biosciences) as per manufacturer’s instructions, and acquired on a BD LSR Fortessa (BD Biosciences). CBA data was analyzed using the FCAP Array Software v3.0 (BD Biosciences).

#### Murine whole tissue RT-qPCR

Pieces of murine spleen and liver were collected into RNA*later* (QIAGEN) and total RNA was extracted using TRIzol Reagent (Invitrogen Life Technologies), RNeasy Cleanup Kit and a RNeasy Mini Kit with on-column DNase digestion (all from QIAGEN) as per the manufacturer’s instructions. RNA samples were reverse transcribed into cDNA using the cDNA Archive Kit (Applied Biosystems, Foster City, CA). RT-qPCR for *Ifng, Tnf and Il10* was performed on CF384 Touch Real-Time PRC Detection System (BIO-RAD) using the TaqMan Gene Expression Assay (Applied Biosciences). Relative quantification was performed using the comparative C_T_ method relative to *Hprt* and *beta2m*.

#### Murine *in vitro* infection

As previously described (Sheel et al., 2015), mice were euthanized by CO_2_ asphyxiation and peritoneal lavage was performed using 5 mL of RT Dulbecco’s Phosphate Buffered Saline (DPBS) (1x) (GIBCO). Peritoneal cells were collected and washed with complete DMEM (10% (v/v) FCS containing 10 mmol _L_-glutamine, 100 mg/ml streptomycin and 100 U/ml penicillin), and 5 × 10^5^ cells were seeded in 16-well glass chamber slides (Lab-Tek, Rochester, NY). Cells were then incubated at 37°C for 24 hours after which non-adherent cells were washed and removed with complete DMEM. LV9 amastigotes were added at a multiplicity of infection (MOI) of 10:1 (in 200 μL complete DMEM). After 1 hour at 37°C, free amastigotes were removed and cells were cultured for another 24 hours with 5 ng/ml recombinant mouse IFNγ (eBioscience, San Diego, CA). On the following day, cells were washed with 1x PBS, fixed and stained. Parasite infectivity was measured as number of parasites per 100 host macrophages.

#### Murine antigen re-stimulation assay

Splenic mononuclear cells were isolated from mice and adjusted to a concentration of 2 × 10^6^ cells/ml. *L. donovani* amastigotes (fixed in 4% PFA) were thawed and washed in RPMI/PS and counted and adjusted to a final concentration of 4 × 10^7^/ml. Cells and parasites were plated into a 96 well U-bottom plate at a 1:20 ratio, where each well contained 1 × 10^5^ cells and 2 × 10^6^ parasites. Cells were cultured with antigen for 72 hours after which cell culture supernatants were collected and IFNγ, TNF and IL-10 cytokine levels were measured using CBA Flex Sets (BD Biosciences) as per manufacturer’s instructions, and acquired on a BD LSR Fortessa (BD Biosciences).

#### Preparation of spleen single cell suspensions

Splenic mononuclear populations were isolated as previously described (Montes de Oca et al., 2016a; Stanley et al., 2008). Briefly, mice were sacrificed by CO_2_ asphyxiation after which the spleen was excised, weighed and collected into 10 mL 2%FBS.PBS (2% (v/v) FBS in 1x PBS). Spleens were passed through a 100 μm cell strainer (Falcon) using the back of a 5 cc/ml syringe plunger (Terumo Medical). Cells were resuspended in media and centrifuged at 350 *g* in an Eppendorf Centrifuge 5810 R (Fisher Scientific, Thermo Fisher Scientific). Cell pellet was incubated in 1 mL Red Blood Cell Lysis Buffer Hybri-Max (Sigma-Aldrich) for 6 minutes at RT. Cells were washed again in 10 mL 2%FBS.PBS after which cell pellet was diluted in DPBS (GIBCO) and Trypan Blue Stain (Invitrogen) and counted using Countess Cell Counting Chamber Slides on the Countess II FL (both from Invitrogen), as per manufacturer’s protocol.

#### Preparation of liver single cell suspensions

Hepatic mononuclear populations were isolated as previously described (Montes de Oca et al., 2016a; Stanley et al., 2008). Briefly, mice were sacrificed by CO_2_ asphyxiation after which the liver was perfused by injecting 5-10 mL of 1x PBS through the portal vein. The liver was excised, weight and collected into 10 mL 2%FBS.PBS. Livers were passed through a 200 μm metal mesh held inside a tea strainer using the back of a 5 cc/ml syringe plunger (Terumo Medical). Mesh was washed with 10-15 mL 2%FBS.PBS and cell suspension was made up to 40 mL and centrifuged at 350 *g* in an Eppendorf Centrifuge 5810 R (Thermo Fisher Scientific). Hepatocytes were separated form leucocytes using a 33% (v/v) Percoll Density Gradient Media (GE Healthcare, Little Chalfond, UK) and centrifuged at 575 *g* for 15 minutes at RT with the brake off. The leucocyte cell pellet was incubated in 1 mL Red Blood Cell Lysis Buffer Hybri-Max (Sigma-Aldrich) for 6 minutes at RT. Cells were washed again in 10 mL 2%FBS.PBS after which cell pellet was diluted in DPBS (GIBCO) and Trypan Blue Stain (Invitrogen) and counted using Countess Cell Counting Chamber Slides on the Countess II FL (both from Invitrogen), as per manufacturer’s protocol.

#### Flow cytometry

Flow cytometry was performed in Falcon® 96-Well Clear Round Bottom Tissue Culture (TC)-Treated Cell Culture Microplates (Corning Inc., Corning NY, USA). Single cell suspensions prepared from various murine organs were incubated with LIVE/DEAD Fixable Aqua Dead Cell Stain Kit (Thermo Fisher) and TruStain fcX (anti-mouse CD16/32; clone: 93; BioLegend, San Diego CA, USA) cocktail for 15 minutes at RT. Cells were washed once with staining buffer (1x PBS, 0.02% (v/v) FBS, 0.01% (w/v) NaN_3_, 5mM EDTA) and centrifuged in an Eppendorf Centrifuge 5810 R (Fisher Scientific, Thermo Fisher Scientific) at 575 *g* for 1 minutes at 4°C. Samples were then incubated with 50 μl of a cocktail of fluorescence-conjugated antibodies toward surface molecules on ice for 20 minutes. After two washes with staining buffer, as described above, samples were incubated with 100 μl of fixation buffer from BD Cytofix Fixation Buffer Set (for cells that are subsequently stained with antibodies against cytokines) (BD Biosciences, San Diego CA, USA) according to manufacturer’s instructions after cells had been incubated in 5 μg/ml brefeldin A (Sigma-Aldrich) in RPMI 1640 medium supplemented with 5% (v/v) FCS for 3 hours at 37°C/5% CO_2_. Cells were then washed twice with wash buffers by centrifuging at 575 *g* for 1 minute at RT, following which, cells were incubated with 50 μl of cocktail containing fluorescence-conjugated antibodies against intracellular molecules for 35 minutes. All staining was performed at RT, and samples were incubated in the dark. A complete list of antibodies can be found in the Key Resources Table. Samples were stored at 4°C before acquisition on a BD LSRFortessa (BD Biosciences) and analyzed on FlowJo software (TreeStar, Ashland, OR, USA).

#### Antibody and drug treatment in mice

CD4^+^ T cells were selectively depleted with 0.5 mg anti-mouse CD4 antibody (clone: GK1.5; BioXCell) administered via intraperitoneal injection every 3 days over a two week period (Bunn et al., 2014). Similarly, neutralization of type I IFN and IFNγ signaling was achieved via administration of 0.1 mg purified anti-mouse IFNAR-1 antibody (clone: MAR1-5A3; Haque et al., 2014) or 0.5 mg anti-mouse IFNγ (clone: XMG1.2; Haque et al., 2011), respectively, every 3 days over a two week period. Control mice received an equivalent amount of rat IgG (Sigma-Aldrich). For anti-parasitic drug treatment experiments, mice were infected for 14 days with *L. donovani* before being treated with either a single, low dose of AmBisome (Amb^lo^; 1 mg/kg) or a single, high dose of AmBisome (Amb^hi^; 10 mg/kg) i.v.. Some mice received 60 mg/kg ruxolitinib (Rux; ChemieTek) orally, twice daily for 5 days, based on dosing used in mice by others (Bhagwat et al., 2014; Xing et al., 2014), or the same volume (200 μl) of vehicle (DMAc; Sigma-Aldrich).

#### Fluorescence Microscopy

Mice were injected with 100 μg i.v. of FITC dextran (Life Technologies) one day before collection of organs. Spleen tissue was collected into 4% PFA and incubated at RT for 1-2 hours before being transferred to a 30% sucrose solution made up in 1x PBS (Sigma-Aldrich) overnight at 4°C. Tissue was then preserved in Tissue-Tek O.C.T. compound (Sakura, Torrance, CA). Splenic architecture and distribution of marginal zone macrophages (MZMs) was performed on 20 μm sections counter-stained with DAPI (1:25000; Sigma-Aldrich) and mounted with Pro-Long Gold anti-fade (Life Technologies). Slides were visualized on an Aperio FL (Leica, Wetzlar, Germany) under 20x magnification. Image analysis was performed using Metamorph 7.8 (Counting App and Region Measurement tool; Integrated Morphometry analysis tool; Molecular Devices, Sunnyvale, CA). The total number of MZMs per mm^2^ was counted in each tissue section.

#### Generation of mixed-BM chimeric mice

Chimeric mice were generated by lethally irradiating recipient mice with two doses of 5.5 Gy (^137^Cs source) and subsequently engrafting with 1x 10^6^ freshly isolated bone marrow cells by i.v. via the lateral tail vein, as previously described (Haque et al., 2011). To examine whether type I IFNs signal directly on T cells, a 90:10 mix of B6.CD45.1/CD45.2 and B6.*Ifnar1^−/−^* (CD45.2) bone marrow cells, respectively, were engrafted into WT recipients, thereby enabling assessment of *Ifnar1^−/−^* CD4^+^ T cell function following priming from a majority of wild-type antigen presenting cells. Mice were maintained on neomycin sulfate (1 g/L; Sigma-Aldrich) for 2 weeks post-engraftment and infected approximately 10-12 weeks thereafter, as previously described (Haque et al., 2011).

#### DC isolation, RT-qPCR and phenotyping

As previously described (Stanley et al., 2008) mice were infected with *L. donovani* and sacrificed 5 hours later. Spleens were digested in collagenase type IV (1 mg/ml; Worthington, Lakewood, NJ) and deoxyribonuclease I (0.5 mg/ml; Worthington) at RT for 45 minutes. Splenocytes were processed as described above. Splenic DCs were isolated from splenocyte preparations by positive selection with anti-CD11c MACS beads, according to the manufacturer’s instructions (Miltenyi Biotec). DCs were also stained for MHCII and CD86 and analyzed by flow cytometry, as described above. Purified DCs were stored in buffer RLT and homogenized in QIAshredder columns (both from QIAGEN). Total RNA was extracted from purified DC using the RNeasy Mini Kit with on-column DNase digestion (both from QIAGEN). RNA samples were reverse transcribed into cDNA using the cDNA Archive Kit (Applied Biosystems, Foster City, CA). RT-qPCR for *Il12* and *Il10* was performed on CF384 Touch Real-Time PRC Detection System (BIO-RAD) using the TaqMan Gene Expression Assay (Applied Bioscience). Relative quantification was performed using the comparative C_T_ method relative to *Hprt* and *beta2m*.

### QUANTIFICATION AND STATISTICAL ANALYSIS

Statistical analysis was performed exclusively in GraphPad Prism 7.02 (GraphPad Software, La Jolla, CA). Analysis of human qPCR and cellular assays was performed using Wilcoxon matched-pairs signed rank test or non-parametric Mann-Whitney tests, as appropriate. Analysis of mouse data used Mann-Whitney tests for comparisons between two groups, and a One-Way ANOVA to assess more than 2 groups within an experiment. p < 0.05 was considered significant. Graphs depict Box and Whisker plots showing minimum and maximum values for human data and mean ± SEM for mouse data, except where individual data points are displayed, in which case only the mean is indicated.

### DATA AND CODE AVAILABILITY

RNA-sequencing data of VL patient CD4^+^ T cells have been deposited in the European Genome-phenome Archive (EGA) database under the accession number EGAS00001004152 (https://www.ebi.ac.uk/ega/studies/EGAS00001004152). This dataset is associated with Figure 1.

## Supplementary Material

Supplementary materials

Supplementary table 1

Supplementary table 2

## Figures and Tables

**Table 1.** Clinical Data for Study Participants

Variables	VL	EC
n	55	18
Age (years)	28.89 ± 15.28 (28)^a^	36.05 ± 10.82 (33.5)
Sex (M/F)	38/17	10/8
Duration of illness (days)	40.18 ± 35.38 (30)	NA
White blood cells (WBC) (× 10^3^/mm^3^, D-0 [day of treatment])	3,714.54 ± 2,084.90 (3,200)	ND
WBC (× 10^3^/mm^3^, D-Dis [day of discharge])	7,130.90 ± 2,291.035 (7,200)	ND
Splenic enlargement (cm, on admission)	3.65 ± 2.81 (4)	NA
Splenic enlargement (cm, at discharge)	0.76 ± 1.59 (0)	NA

NA, not applicable; ND, not done.

a Mean values ± SD of aggregated data are shown, and median values are shown in parentheses.

**Table:** KEY RESOURCES TABLE

REAGENT or RESOURCE	SOURCE	IDENTIFIER
Antibodies		
Alexa Fluor® 488 anti-mouse FOXP3 (clone: MF-14)	BioLegend	Cat#126406, RRID:AB_1089113
Alexa Fluor® 647 Anti-Mouse/Human FOXP3 (clone: 3G3)	eBioscience	Cat#MA5-18160, RRID:AB_2539534
Alexa Fluor® 700 anti-mouse CD44 (clone: IM7)	BioLegend	Cat#103026, RRID:AB_493713
Alexa Fluor® 700 anti-mouse CD45.1 (clone: A20)	BioLegend	Cat#110724, RRID:AB_493733
Alexa Fluor® 700 anti-mouse CD8a (clone: 53-6.7)	BioLegend	Cat#100730, RRID:AB_493703
Alexa Fluor® 700 anti-mouse TCR b chain (clone: H57-597)	BioLegend	Cat#109224, RRID:AB_1027648
Anti-Human Interferon Alpha/Beta Receptor Chain 2 mAb (clone MMHAR-2)	R&D Systems	Cat#21385-1, RRID:AB_354167
Anti-Mouse IFNAR-1 Purified (clone: MAR1-5A3)	Leinco Technologies	Cat#I-400, RRID: N/A
APC anti-mouse CD11c (clone: N418)	BioLegend	Cat#117310, RRID:AB_313779
APC anti-mouse IFN-g (clone: XMG1.2)	BioLegend	Cat#505810, RRID:AB_315404
APC anti-mouse IL-10 (clone: JES5-16E3)	BioLegend	Cat#505010, RRID:AB_315364
APC anti-T-bet antibody (clone: 4B10)	BioLegend	Cat#644814, RRID:AB_10901173
APC-conjugated I-A^b^-PEPCK_335-351_ tetramer	NIH Tetramer Core Facility	N/A
APC/Cy7 anti-mouse CD4 (clone: RM4-5)	BioLegend	Cat#100526, RRID:AB_312727
APC/Cy7 anti-mouse NK1.1 (clone: PK136)	BioLegend	Cat#108724, RRID:AB_830871
BD Horizon BUV395 Rat Anti-Mouse CD4 (clone: GK1.1)	BD Biosciences	Cat#563790, RRID:AB_2738426
BD Horizon BUV737 Hamster Anti-Mouse TCR b Chain (clone: H57-597)	BD Biosciences	Cat# 612821, RRID:N/A
BD Horizon BV395 Mouse Anti-Mouse CD45.2 (clone: 104)	BD Biosciences	Cat#564616, RRID:AB_2738867
Brilliant Violet 421 anti-mouse I-A/I-E (clone: M5/114.15.2)	BioLegend	Cat#107631, RRID:AB_10900075
Brilliant Violet 605 anti-mouse Ly-6C (clone: HK1.4)	BioLegend	Cat#128036, RRID:AB_2562353
Brilliant Violet 650 anti-mouse TNF-a (clone: MP6-XT22)	BioLegend	Cat#506333, RRID:AB_2562450
Brilliant Violet 711 anti-mouse CD11c (clone: N418)	BioLegend	Cat#117349, RRID:AB_2563905
Brilliant Violet 421 anti-mouse TCR b chain (clone: H57-597)	BioLegend	Cat#109229, RRID:AB_10933263
FITC anti-mouse CD11a (clone: M17/4)	BioLegend	Cat#101106, RRID:AB_312779
FITC anti-mouse CD11a (clone: M17/4)	BioLegend	Cat#101106, RRID:AB_312779
FITC anti-mouse CD45.2 (clone: 104)	BioLegend	Cat#109806, RRID:AB_313443
FITC anti-mouse/human CD44 (clone: IM7)	BioLegend	Cat#103006, RRID:AB_312957
*InVivo*mAb anti-mouse CD4 (clone: GK1.1)	BioXCell	Cat#BE0003-1, RRID:AB_1107636
*InVivo*mAb anti-mouse IFNγ (clone: XMG1.2)	BioXCell	Cat#BP0055, RRID:AB_1107694
Mouse IgG_2A_ Isotype Control (clone: 20102)	R&D Systems	Cat#MAB003, RRID:AB_357345
PE anti-mouse IL-10 (clone: JES5-16E3)	BioLegend	Cat#505008, RRID:AB_315362
PE CD86 (clone: GL-1)	BioLegend	Cat#105008, RRID:AB_313151
PE/Cy7 anti-mouse CD49d (clone: R1-2)	BioLegend	Cat#103618, RRID:AB_2563700
PE/Cy7 anti-mouse IFN-g (clone: XMG1.2)	BioLegend	Cat#505826, RRID:AB_2295770
PE/Cy7 anti-mouse Ly-6G (clone: 1A8)	BioLegend	Cat#127618, RRID:AB_1877261
PE/Cy7 anti-mouse TNF-a (clone: MP6-XT22)	BioLegend	Cat#506324, RRID:AB_2256076
PE/Dazzle 594 F4/80 (clone: BM8)	BioLegend	Cat#123146, RRID:AB_2564133
PerCP/Cyanine5.5 anti-mouse CD4 (clone: RM4-5)	BioLegend	Cat#100540, RRID:AB_893326
PerCP/Cyanine5.5 anti-mouse/human CD11b (clone: M1/70)	BioLegend	Cat#101228, RRID:AB_893232
Ultra-LEAF Purified anti-human HLA-DR (clone: L243)	BioLegend	Cat#307648, RRID:AB_2561493
Ultra-LEAF Purified Mouse IgG2a, _K_ Isotype Control Antibody (clone: MOPC-173)	BioLegend	Cat#400264, RRID:AB_11148947
Bacterial and Virus Strains		
*L. donovani* amastigotes	Clinical isolates from VL patients at Kala-azar Medical Research Center, Muzaffapur, Bihar, India	N/A
*L. donovani* amastigotes (LV9; MHOM/ET/67/HU3)	Bradley and Kirkley (1977)	N/A
Biological Samples		
PBMCs from healthy endemic controls	Kala-azar Medical Research Center, Muzaffapur, Bihar, India	N/A
PBMCs from visceral leishmaniasis patients	Kala-azar Medical Research Center, Muzaffapur, Bihar, India	N/A
Chemicals, Peptides, and Recombinant Proteins		
2-Mercaptoethanol	Sigma-Aldrich	Cat#M6250
Amphotericin liposomal 50 mg powder for injection, AmBisome®	Gilead Sciences	N/A
BD Cytofix Fixation Buffer Set	BD Biosciences	Cat#554714
Brefeldin A	Sigma-Aldrich	Cat#6542
DAPI for nucleic acid staining	Sigma-Aldrich	Cat#D9542
Dextran, Fluorescein	Life Technologies	Cat#D7137
eBioscience Foxp3/Transcription Factor Staining Buffer Set	Thermo Fisher Scientific	Cat#00-5523-00
EDTA	Amresco	Cat#E177-500ML
Ficoll (Lymphoprep)	Stem Cell	Cat#7861
Giemsa stain, modified	Sigma-Aldrich	Cat#GS500
Medium 199, Hanks’ Balanced Salts	Thermo Fisher	Cat#12350039
Monensin Solution (1,000X)	BioLegend	Cat#420701
Neomycin trisulfate salt hydrate powder	Sigma-Aldrich	Cat#N1876-100G
N,N-Dimethylactamide anhydrous	Sigma-Aldrich	Cat#271012-100ML
N-acetyl-L-leucyl-L-leucyl-L-argininal	Amresco	Cat#J580-5MG
Percoll density gradient media	GE Healthcare	Cat#17089101
Phenylmethanesulphonyl fluoride (PMSF)	HiMedia	Cat#RM1592
Phorbol 12- myristate 13-acetate	Sigma-Aldrich	Cat#P8139
ProLong Gold Antifade	Invitrogen	Cat#P36934
Recombinant Mouse IFN-gamma Protein	R&D Systems	Cat#485-MI
Red blood cell lysing buffer Hybri-Max	Sigma-Aldrich	Cat#R7757
RNA*later*	Invitrogen	Cat#AM7021
Ruxolitinib	ChemieTek	Cat#CT-INCB
Ruxolitinib, 5 mg	Santa Cruz Biotechnology	Cat#sc-364729
Tissue-Tek® O.C.T. Compound	Sakura	Cat#4583
Trizma® hydrochloride solution (TRIS-HCl)	Sigma-Aldrich	Cat#T2444
Trypan blue stain (0.4%) for use with Countess Automated Cell Counter	Invitrogen	Cat#T10282
Zombie Aqua fixable viability dye kit	BioLegend	Cat#423102
Critical Commercial Assays		
Agencourt AMPure XP	Beckman Coulter	Cat#A63881
BD Cytometric Bead Array – Mouse IFN-γ Flex Set	BD Biosciences	Cat#558296
BD Cytometric Bead Array – Mouse IL-10 Flex Set	BD Biosciences	Cat#558300
BD Cytometric Bead Array – Mouse TNF Flex Set	BD Biosciences	Cat#558299
DNA 1000 Kit	Agilent	Cat#5067-1504
DNase Max Kit (50)	QIAGEN	Cat#15200-50
ELISA MAX Deluxe Set Human IFNγ	BioLegend	Cat#430104
High-capacity cDNA Reverse Transcription Kit	Thermo Fisher	Cat#4368814
LS Columns	Miltenyi Biotec	Cat#130-042-401
MACS CD1c (BDCA-1)^+^ Dendritic Cell Isolation Kit, human	Miltenyi Biotec	Cat#130-119-475
MACS CD4 MicroBeads, human	Miltenyi Biotec	Cat#130-045-101
MACS CD11c MicroBeads UltraPure, mouse	Miltenyi Biotec	Cat#130-108-338
MACS CD14 MicroBeads, human	Miltenyi Biotec	Cat#130-050-201
MACS CD19 MicroBeads, human	Miltenyi Biotec	Cat#130-050-301
MS Columns	Miltenyi Biotec	Cat#130-042-201
NextSeq 500/550 High Output Kit v2 (150 cycles)	Illumina	Cat#FC-404-2002
QIAshredder (50)	QIAGEN	Cat#79654
Qubit DNA HS Assay Kit	Life Technologies	Cat#Q32854
Qubit RNA HS Assay Kit	Thermo Fisher	Cat#Q32852
RNA 6000 Nano kit	Agilent Technologies	Cat#5067-1511
RNase-Free DNase Set (50)	QIAGEN	Cat#79254
RNeasy Mini Kit (50)	QIAGEN	Cat#74104
Superscript III Reverse Transcriptase	Life Technologies	Cat#18080044
TruSeq Stranded mRNA Library Prep Kit High Throughput (96 samples, 96 indexes)	Illumina	Cat#RS-122-2103
Deposited Data		
Peripheral blood CD4^+^ T cells isolated from *L. donovani*-infected patients, RNA-seq data	This paper	European Genome-phenome Archive (EGA) database accession number EGAS00001004152
Experimental Models: Organisms/Strains		
B6.129P2-*Lyz2^tm1(cre)lfo^*/J	Bred in house; Clausen et al. (1999)	RRID: IMSR_JAX:004781
B6.129S4-*Ccr2^tm1lfc^*/J	Bred in house; Boring et al. (1997)	RRID: IMSR_JAX:004999
B6.129S7-*Rag1^tm1Mom^*/J	Bred in house; Mombaerts et al. (1992)	RRID: IMSR_JAX:002216
B6.(Cg)-*Ifnar1^tm1.1Ees^*/J	Bred in house; Prigge et al. (2015)	RRID: IMSR_JAX:028256
B6.(Cg)-*Ifnar1^tm1.2Ees^*/J	Bred in house; Prigge et al. (2015)	RRID: IMSR_JAX:028288
B6.*Ifnar1*^ΔDC^	Bred in house; This paper.	N/A
B6.*Ifnar1*^ΔLysM^	Bred in house; This paper.	N/A
B6.Cg-Tg(Itgax-cre)1-1Reiz/J	Bred in house; Caton et al. (2007)	RRID: IMSR_JAX:008068
B6.SJL-*Ptprc^a^Pepc^b^*/BoyJArc	Australian Resource Centre	RRID: IMSR_JAX:002014
C57BL/6JArc	Australian Resource Centre	RRID: IMSR_ARC:B6
Oligonucleotides		
Eukaryotic 18S rRNA Endogenous Control (VIC/MGB probe, primer limited)	Thermo Fisher	Cat#:4319413E
TaqMan Gene Expression Assay (FAM MGB) – human *IFNB1* (Hs01077958_s1), human *IFNA1* (Hs00855471_g1), human *IFNAR1* (Hs01066118_m1), human *IFNAR2* (Hs01022060_m1).	Thermo Fisher	Cat#4331182
TaqMan MGB Probe - mouse *Il12p40* (Mm00434174_m1), mouse *Tnf* (Mm0041889_m1), *Il10* (Mm01288386_m1), *Hprt* (Mm01318743_m1), *b2m* (Mm00437762_m1)	Applied Biosciences	Cat#4331182
Software and Algorithms		
Adobe Illustrator, V23.0.3	Adobe. https://www.adobe.com/	N/A
Cutadapt, v1.11	Martin, 2011	N/A
EdgeR	Robinson et al. (2010) https://bioconductor.org/packages/release/bioc/html/edgeR.html	N/A
FACSDiva, v8.0	BD Biosciences https://www.bdbiosciences.com/en-us/instruments/research-instruments/research-software/flow-cytometry-acquisition/facsdiva-software	N/A
FCAP Array Software, v3.0	BD Biosciences. https://www.bdbiosciences.com/us/applications/research/bead-based-immunoassays/analysis-software/fcap-array-software-v30/p/652099	Cat#652099
FlowJo, v10 OSX	FlowJo, LLC. https://www.flowjo.com/	N/A
GraphPad Prism 7, v7.0c	GraphPad Software. https://www.graphpad.com/scientific-software/prism/	N/A
Ingenuity Pathway Analysis (IPA), winter 2018 release	QIAGEN https://digitalinsights.qiagen.com/products-overview/discovery-insights-portfolio/content-exploration-and-databases/qiagen-ipa/	N/A
R Project for Statistical Computing	https://www.r-project.org/	RRID: SCR_001905
RNA-SeQC, v1.1.8	DeLuca et al. (2012)	N/A
RSEM, V1.2.30	Li and Dewey (2011)	N/A
RStudio Desktop, V1.0.136 - 1.2.1335	https://rstudio.com/products/RStudio/	N/A
Spliced Transcripts Alignment to a Reference (STAR), v2.5.2a	Dobin et al. (2013) https://github.com/alexdobin/STAR	N/A
Other		
Ensembl (release 89) gene model		N/A
GRCh37 assembly	https://m.ensembl.org/info/website/tutorials/grch37.html	N/A

## Highlights

- Type I IFNs are major upstream regulators of CD4^+^ T cells from VL patients
- Type I IFN signaling-deficient mice have improved control of *Leishmania donovani*
- Type I IFNs inhibit IFNγ but promote IL-10-producing antigen-specific CD4^+^ T cells
- Blocking type I IFN signaling enhances anti-parasitic CD4^+^ T cell responses
