# Supplementary material for: Type I Interferons Suppress Anti-parasitic Immunity and Can Be Targeted to Improve Treatment of Visceral Leishmaniasis
Source: Cell Rep. Author manuscript; Available in PMC 2021 Mar 21. (PMC7981274; doi:10.1016/j.celrep.2020.01.099)
Supplement: Supplementary materials [file NIHMS1668495-supplement-Supplementary_materials.pdf]

Cell Reports, Volume *30*

Supplemental Information

Type I Interferons Suppress Anti-parasitic Immunity and Can Be Targeted to Improve Treatment of Visceral Leishmaniasis

Rajiv Kumar, Patrick T. Bunn, Siddharth Sankar Singh, Susanna S. Ng, Marcela Montes de Oca, Fabian De Labastida Rivera, Shashi Bhushan Chauhan, Neetu Singh, Rebecca J. Faleiro, Chelsea L. Edwards, Teija C.M. Frame, Meru Sheel, Rebecca J. Austin, Steven W. Lane, Tobias Bald, Mark J. Smyth, Geoffrey.R. Hill, Shannon E. Best, Ashraful Haque, Dillon Corvino, Nic Waddell, Lambross Koufariotis, Pamela Mukhopadhay, Madhukar Rai, Jaya Chakravarty, Om Prakash Singh, David Sacks, Susanne Nylen, Jude Uzonna, Shyam Sundar, and Christian R. Engwerda

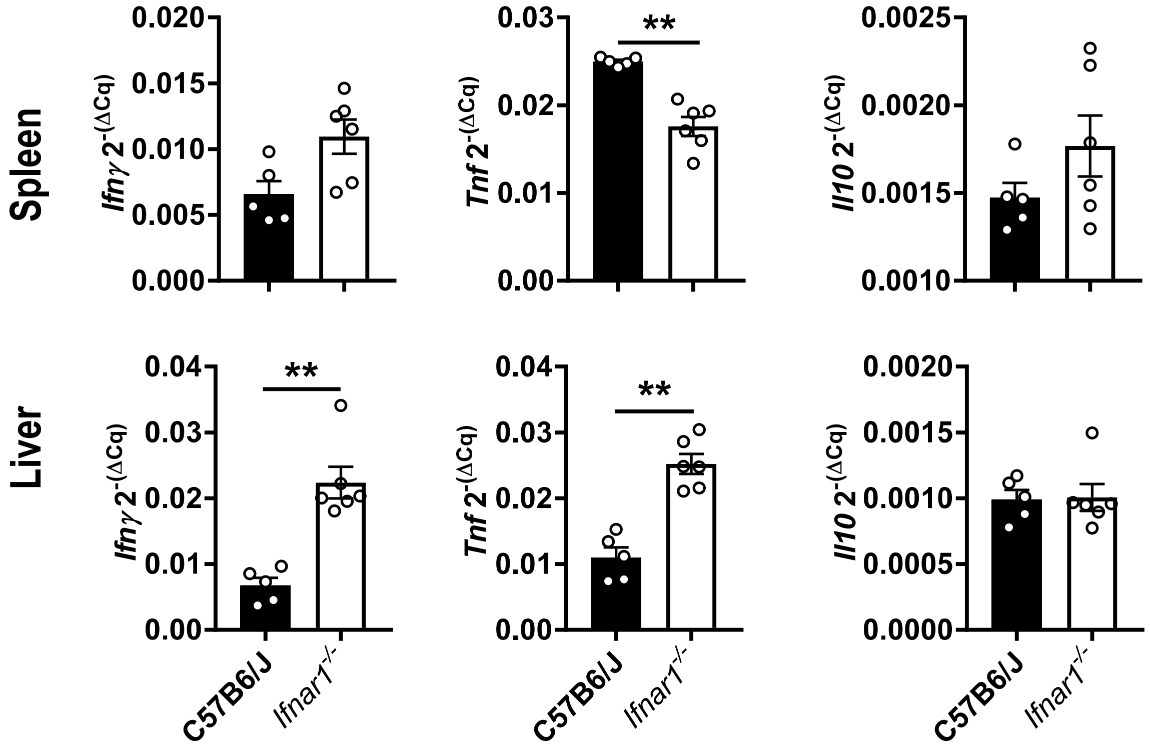

Supplementary Figure 1. *Ifnar1*-deficient mice have increased *Ifng* and *Tnf* mRNA in the liver. Related to Figure 3. RNA was isolated from spleen and liver tissue from *L. donovani*-infected *Ifnar1*$^{-/-}$ (open columns) and control B6 WT (closed columns) mice at day 14 p.i., and *Ifng*, *Tnf* and *Il10* mRNA measured by qPCR. n = 5-6 mice per group. The experiment was conducted once. Mean ± SEM, *p<0.05; significance assessed by Mann-Whitney tests.

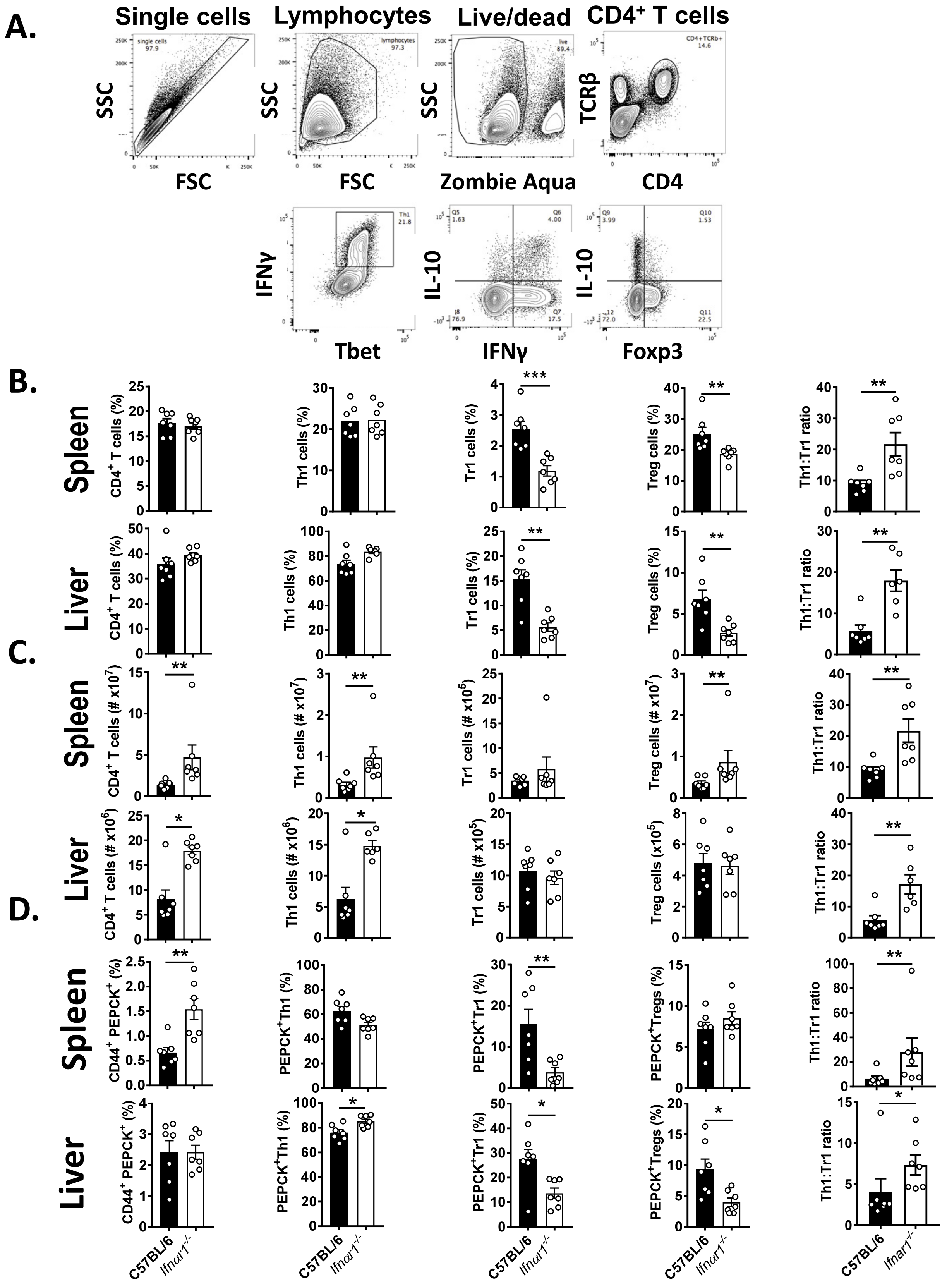

Supplementary Figure 2. ***Ifnar1*-deficient mice have improved CD4⁺ T cell responses.** Related to Figure 4. Cells were isolated from mice 14 days after infection with *L. donovani* and subjected to FACS staining. Single cells were selected, followed by lymphocytes and then live cells. CD4⁺ T cells were gated based on TCRβ and CD4 and then Th1 cells were identified based on IFNγ and Tbet expression, Tr1 cells based on IL-10 and IFNγ co-production and Treg cells on Foxp3 expression (A). *Ifnar1*$^{-/-}$ (open columns) and control B6 WT (closed columns) mice were infected with *L. donovani* for 14 days, prior to measuring polyclonal CD4⁺ T cell frequency (B) and number (C) in spleen and liver, as indicated. The frequency of activated PEPCK⁺ CD4⁺ T cells and Th1 cells , Tr1 cells and Treg cells in the spleen and liver, as indicated, is also shown (D). n=7 mice per group. Experiment conducted 3 times. Mean ± SEM, *p<0.05, **p<0.01 and ***p<0.001; significance assessed by Mann-Whitney tests.

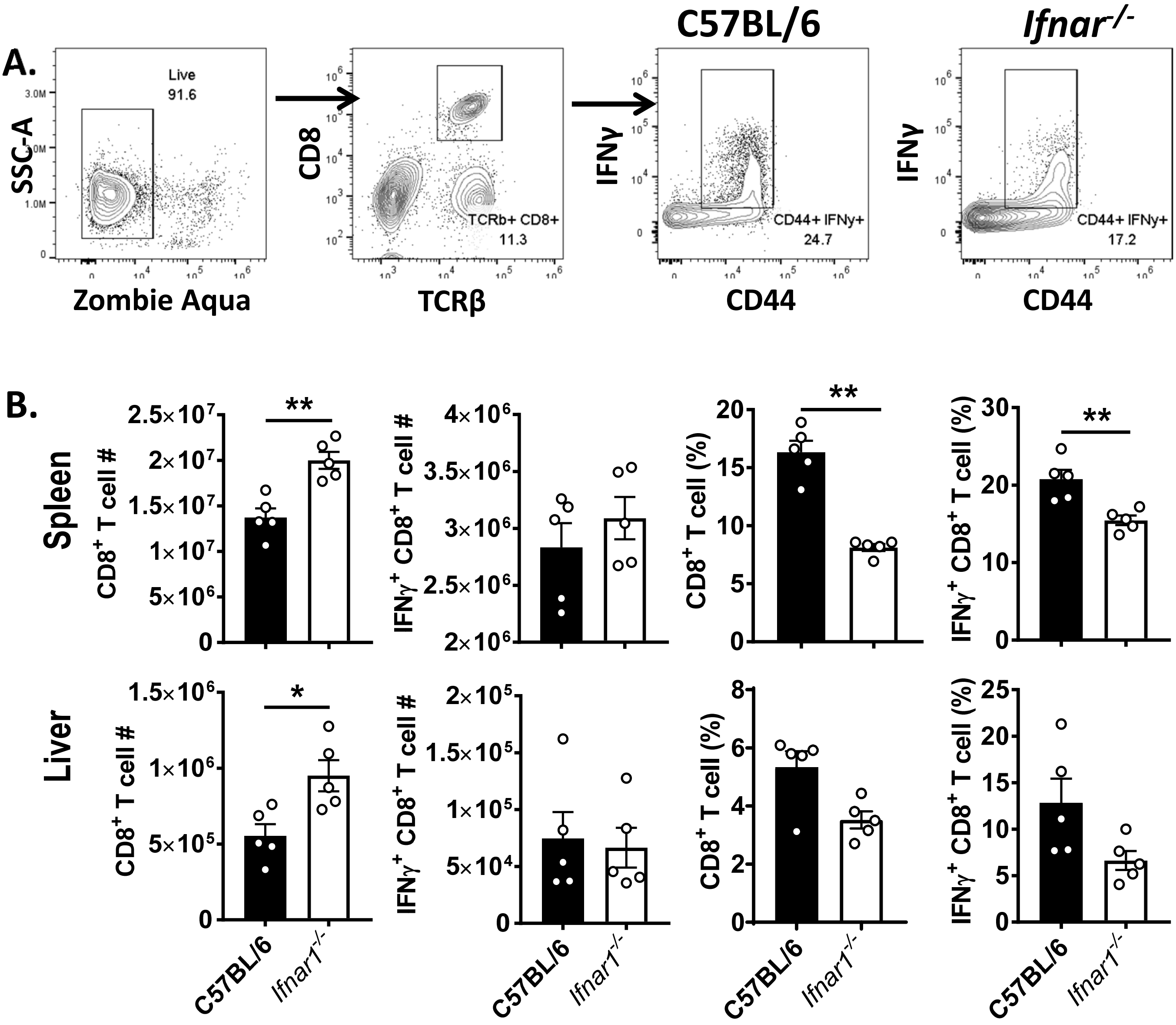

Supplementary Figure 3. CD8⁺ T cell responses in *Ifnar1*-deficient mice. Related to Figure 4. Live lymphocytes were gated as shown in Supplementary Figure 2, and then gated on CD8⁺ T cells (A). *Ifnar1*$^{-/-}$ (open columns) and control B6 WT (closed columns) mice were infected with *L. donovani* for 14 days, prior to measuring polyclonal CD8⁺ T cell and IFNγ CD8⁺ T cell number and frequency in spleen and liver, as indicated (B). Experiment conducted 3 times. Mean ± SEM, *p<0.05 and **p<0.01; significance assessed by Mann-Whitney tests.

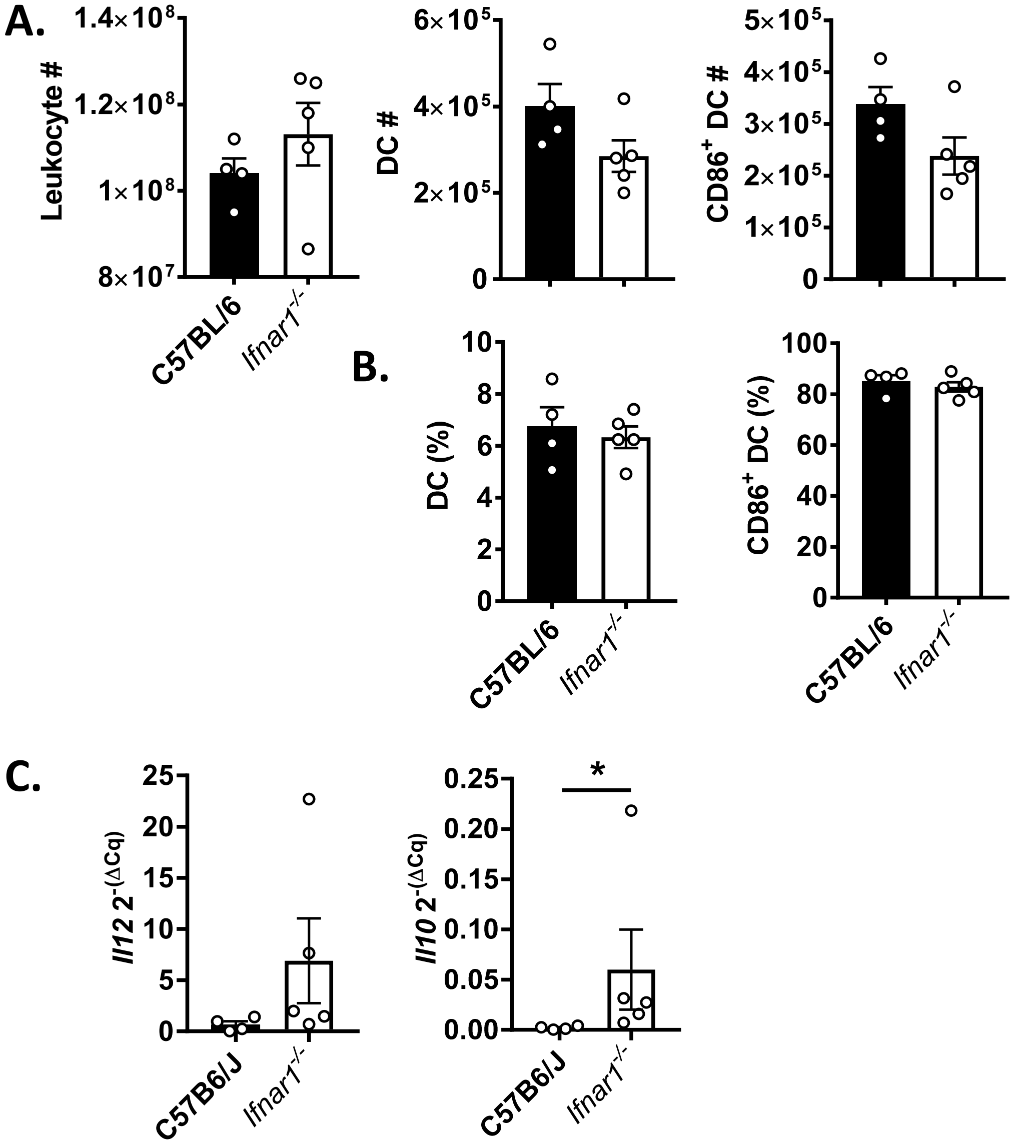

Supplementary Figure 4. DC activation in the spleen of *Ifnar1*-deficient mice. Related to Figure 6. Live lymphocytes were gated as shown in Supplementary Figure 2, and then gated on DCs as shown in Supplementary Figure 6. *Ifnar1*$^{-/-}$ (open columns) and control B6 WT (closed columns) mice were infected with *L. donovani* for 5 hours, prior to measuring leukocyte, DC and CD86^{+} DC number in the spleen (A). The frequency of DCs and CD86^{+} DCs in the spleen was also measured (B). Splenic CD11c^{+} cells were also isolated and mRNA purified to measure *Il12* and *Il10* mRNA by qPCR (C). Experiment conducted once. Mean ± SEM, *p<0.05; significance assessed by Mann-Whitney tests.

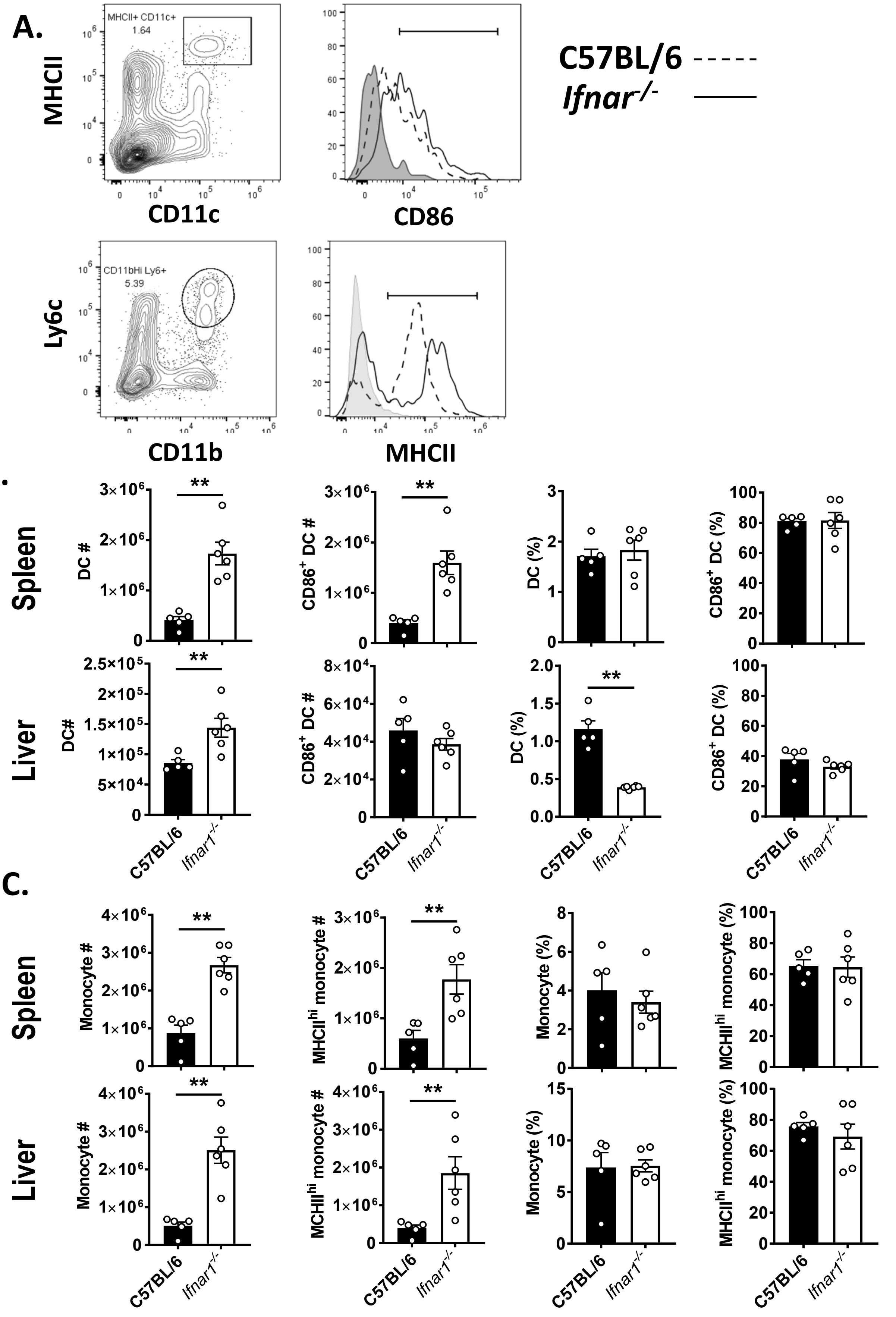

Supplementary Figure 5. DC and monocyte responses in *Ifnar1*-deficient mice. Related to Figure 6. Live lymphocytes were gated as shown in Supplementary Figure 2, and then gated on DCs and monocytes (A). *Ifnar1*$^{-/-}$ (open columns) and control B6 WT (closed columns) mice were infected with *L. donovani* for 14 days, prior to measuring DC and CD86^{+} DC number and frequency in spleen and liver, as indicated (B). Monocyte and activated monocyte (MHCII^{hi}) number and frequency in spleen and liver were also measured, as indicated (C). Experiment conducted 3 times. Mean ± SEM, **p<0.01; significance assessed by Mann-Whitney tests.

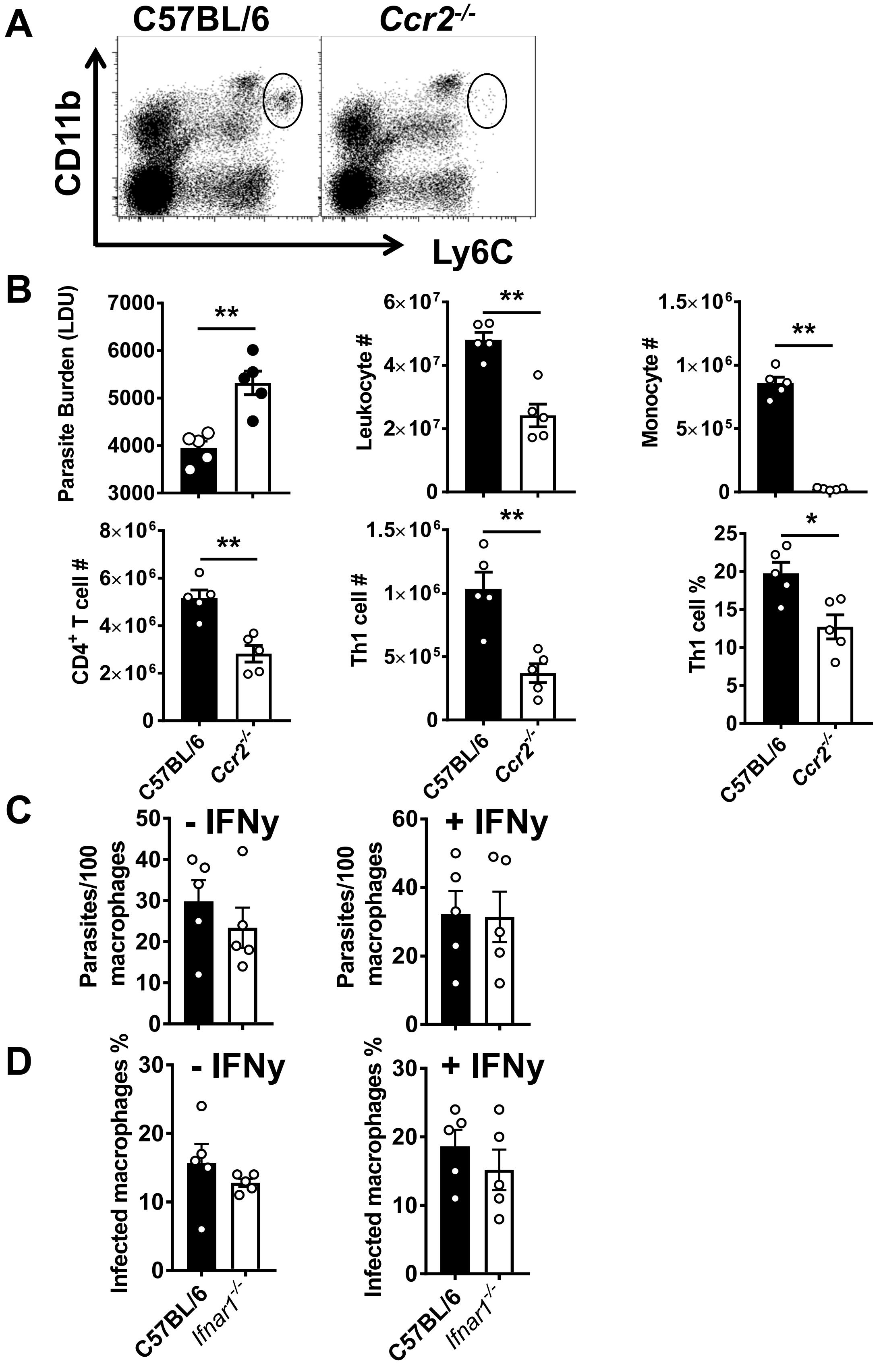

Supplementary Figure 6. Th1 cell responses in *Ccr2*-deficient mice. Related to Figure 6. Live lymphocytes were gated as shown in Supplementary Figure 2, and then gated on monocytes (A). *Ccr2*$^{-/-}$ (open columns) and control B6 WT (closed columns) mice were infected with *L. donovani* for 14 days, prior to measuring liver parasite burden, leukocyte, monocyte, $CD4^+$ T cell and Th1 cell number and Th1 cell frequency, as indicated (B). Experiment conducted 3 times. Mean ± SEM, *p<0.05 and **p<0.01; significance assessed by Mann-Whitney tests. Live lymphocytes were gated as shown in Supplementary Figure 2, and then gated on monocytes (C). *Ccr2*$^{-/-}$ (open columns) and control B6 WT (closed columns) mice were infected with *L. donovani* for 14 days, prior to measuring liver parasite burden, leukocyte, monocyte, $CD4^+$ T cell and Th1 cell number and Th1 cell frequency, as indicated (D). Experiment conducted 3 times. Mean ± SEM, *p<0.05 and **p<0.01; significance assessed by Mann-Whitney tests.

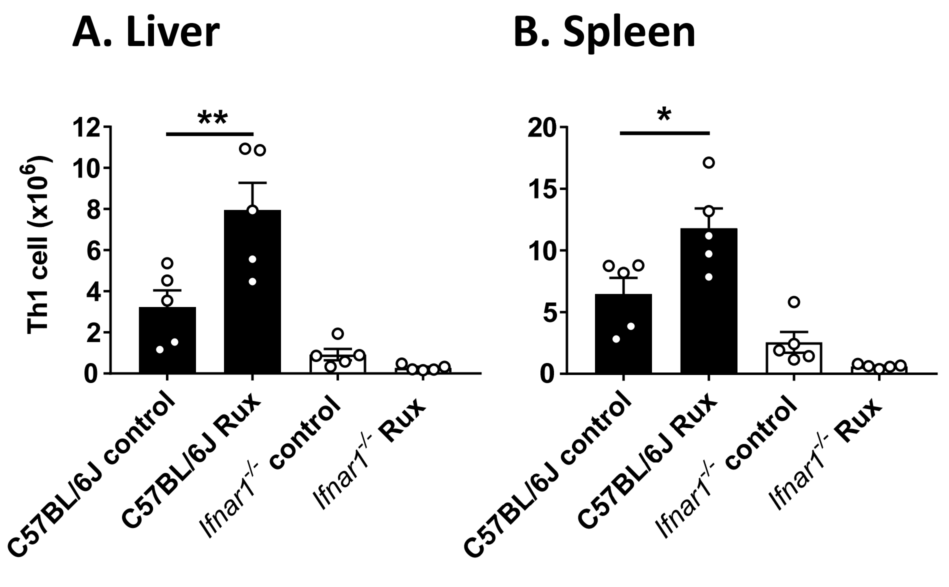

Supplementary Figure 7. Type I interferon signaling blockade increased polyclonal CD4$^+$ T cell responses. Related to Figure 7. Polyclonal CD4$^+$ T cell responses were measured using the same gating strategy shown in Figure 5A at day 28 p.i., as indicated. n=4-7 mice per group. Experiment conducted twice. Mean ± SEM, *p<0.05 and **p<0.01; significance assessed by one-way ANOVA.
