# Supplementary material for: Type I Interferons Suppress Anti-parasitic Immunity and Can Be Targeted to Improve Treatment of Visceral Leishmaniasis
Source: Cell Rep. Author manuscript; Available in PMC 2021 Mar 21. (PMC7981274; doi:10.1016/j.celrep.2020.01.099)
Supplement: Supplementary table 1 [file NIHMS1668495-supplement-Supplementary_table_1.docx]

| Variables | VL | EC |
| --- | --- | --- |
| N | 55 | 18 |
| Age (years) | 28.89±15.28 (28)^a^ | 36.05±10.82 (33.5) |
| Sex (M/F) | 38/17 | 10/8 |
| Duration of illness (days) | 40.18±35.38 (30) | N/A |
| WBC (×10^3^/mm^3^, D-0) | 3714.54±2084.90 (3200) | N/D |
| WBC (×10^3^/mm^3^, D-Dis) | 7130.90±2291.035 (7200) | N/D |
| Splenic enlargement  (cm, on admission) | 3.65±2.81 (4) | N/A |
| Splenic enlargement  (cm, at discharge) | 0.76±1.59 (0) | N/A |

**Table 1. Clinical data for study participants.**

Abbreviations: N/A, not applicable; ND, not done.

^a^Mean values ± SD of aggregated data are shown, and median values are in parentheses.
